# Single-cell multi-omics analysis decodes molecular characteristics of sheep oocyte fate *in vivo* maturation

**DOI:** 10.1016/j.fmre.2025.10.009

**Published:** 2025-12-08

**Authors:** Yujun Yao, Zihuan Du, Qiang Zhang, Xiaochen Kou, Hongyu Yang, Chanyuan Pan, Jing Li, Xiangwei Sun, Lu Zhang, Guoshi Liu, Xiaoling Xu, Shuai Gao

**Affiliations:** a Frontiers Science Center for Molecular Design Breeding (MOE), State Key Laboratory of Animal Biotech Breeding, College of Animal Science and Technology, China Agricultural University, Beijing 100193, China; b State Key Laboratory of Female Fertility Promotion, Key Laboratory of Assisted Reproduction (Peking University), Ministry of Education, Beijing Key Laboratory of Reproductive Endocrinology and Assisted Reproductive Technology, Center for Reproductive Medicine, Department of Obstetrics and Gynecology, Peking University Third Hospital, Beijing 100191, China; c Institute of Animal Husbandry and Veterinary Medicine, Beijing Academy of Agriculture and Forestry Sciences, Beijing 100097, China; d Institute of Cardiovascular Diseases, Xiamen Cardiovascular Hospital, School of Medicine, Xiamen University, Xiamen 361006, China; e College of Animal Science &Veterinary Medicine, Shenyang Agricultural University, Shenyang 110866, China; f College of Food Science and Nutritional Engineering, China Agricultural University, Beijing 100083, China

**Keywords:** Sheep, *in vivo*, Oocyte, Antral follicle, Single-cell multi-omics technology

## Abstract

A competent oocyte in mammals undergoes a remarkably elaborate process during follicle growth. Oocytes in antral follicles of varying sizes are subject to the potential outcomes of maturation. However, the comprehensive multi-molecular mechanisms underlying oocyte development fate during antral folliculogenesis remain largely elusive. Here, we employed sheep, a large non-primate species of economic importance, as the study model. Oocytes and granulosa cells from antral follicles of varying sizes were collected and then analyzed with our modified single-cell multi-omics sequencing technology to profile their transcriptomes, DNA methylation patterns, and chromatin accessibility features. The *in vivo* matured metaphase II (MII) oocytes were utilized to infer the fate trajectories of different oocytes. Transcriptomic profiling unveiled three distinct oocyte subtypes (Type 1, 2 and 3), with Type 3 oocytes identified as those progressing towards a maturation-competent fate. DNA methylation and chromatin accessibility data also revealed distinct differences among those three oocyte types. A detailed analysis of Type 3 oocytes revealed that, while preparing key factors for maternal RNA degradation (M-decay), those oocytes accomplished the majority of maternal RNA clearance. Moreover, cell-cell communication analysis between oocytes and granulosa cells revealed a high enrichment of the Endothelin 1 (ET-1) signaling pathway in Type 3 oocytes. Further experimental validation showed that adding ET-1 enhanced the function of important organelles, such as mitochondria, endoplasmic reticulum, Golgi apparatus, and cortical granules in mature oocytes, facilitating spindle assembly and thereby improving oocyte developmental competence. Conversely, ET-1 receptor antagonists (BQ123) led to a decline in oocyte quality. Notably, ET-1-treated oocytes exhibited elevated blastocyst formation rates post in vitro fertilization (IVF). In summary, our study provides new insights into the orchestration mechanisms governing oocyte fate determination in mammals and offers a novel strategy for enhancing sheep oocyte quality and developmental potential during in vitro maturation.

## Graphical abstract

**Figure:**
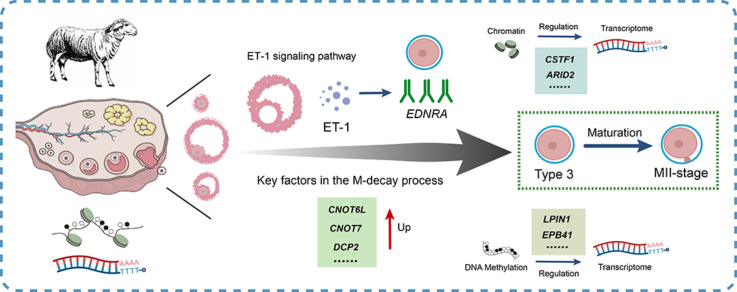

## 1 Introduction

Mammalian life originates from the exquisite biological process of the union between male and female gametes [1]. The female gamete, namely the oocyte, carries the entire genetic information from the maternal parent, and within its cytoplasm, harbors a substantial number of substances that ensure the normal development of the early zygote [2]. The generation and maturation of oocytes involve several critical stages: the formation of primordial germ cells (PGCs), oocyte differentiation, activation and development of follicular structures encapsulating oocytes, and the eventual maturation and ovulation of oocytes [3]. Of note is that not all oocytes can smoothly progress through the process of maturation and ovulation; most follicles containing oocytes will gradually undergo atresia [3]. The growth of follicles is concomitant with the gradual maturation of oocytes, and larger follicles are morphologically more likely to achieve maturation and ovulation [4]. Furthermore, whether oocytes acquire complete developmental competence directly influences the rate of blastocyst formation following fertilization [5]. Hence, dissecting the molecular mechanisms that determine whether oocytes acquire complete developmental competence during their development holds profound implications, for both enhancing livestock reproductive capabilities and optimizing assisted reproductive technologies for clinic.

Sheep, has been raised as a livestock of significant economic value, undergo a process of in vitro embryo production where oocytes are selected for in vitro maturation (IVM) and in vitro fertilization based on the size of antral follicles [6]. However, only 20−50% of oocytes can successfully mature in vitro and develop into blastocysts following fertilization [7]. A substantial part of this limitation stems from the incomplete understanding of the molecular mechanisms governing the maturation process of sheep oocytes from antral follicles to ovulation [8]. Consequently, deciphering this intricate process is crucial for enhancing the efficiency of in vitro embryo production in sheep.

Previous studies have indicated that the diameter of sheep antral follicles increases gradually from < 3 mm to over 6 mm during the development [9]. Furthermore, in the IVM process of oocytes in sheep, oocytes collected from larger follicles exhibit a higher non-atresia rate [10]. A study utilizing laser capture microdissection technology and microarrays described the gene expression patterns of oocytes at different stages within sheep follicles [11]. Based on transcriptomic data from the ovaries at different developmental stages of sheep, genes associated with follicle growth were identified [12]. The development of single-cell sequencing technology has made it possible to unravel the gene characteristics of oocytes in different species [[13], [14], [15], [16]]. For instance, previous studies have elucidated the transcriptional regulatory features during human follicular development [13]. The maturation of single-cell multi-omics sequencing has enabled the description of the epigenetic characteristics of mouse oogenesis as well as human oogenesis [14,15]. More importantly, using our modified single-cell multi-omics technologies, we have reported the regulatory networks in bovine oocyte fate determination during antral follicle growth [17]. During the development of oocytes in sheep, researchers have delineated the corresponding molecular networks and metabolic characteristics through single-cell transcriptomics and ultra-sensitive metabolomics [18,19]. These studies provide valuable insights into the maturation process of sheep oocytes. However, it was still insufficient for a comprehensive understanding of the complex biological process of oocyte development *in vivo*. The integration of omics approaches is urgently needed to understand sophisticated mechanisms involved in governing the fate-determining processes in the maturation of sheep oocytes.

In this study, we aim to elucidate the molecular characteristics that determine oocytes’ complete developmental competence in the process of ovine antral follicle growth. Here, we applied the modified single-cell multi-omics sequencing technology to simultaneously analyze the gene expression profiles, DNA methylation, and chromatin accessibility of oocytes and granulosa cells (GCs) from antral follicles of different sizes in sheep [[20], [21], [22]]. This approach delineated the multi-omics molecular features associated with various developmental fates of sheep oocytes. According to our systematic analysis results, we further investigated the important role of EDN (Endothelin) signaling in the developmental potential of sheep oocytes. These findings may provide important insights for in vitro maturation of oocytes and subsequent embryo production.

## 2 Materials and methods/experiment

### 2.1 Collection and culture of the cumulus-oocyte complexes (COCs)

Ovaries collected from healthy adult ewes at a slaughterhouse were stored in 37 °C physiological saline (NaCl (0.9%, Sigma-Aldrich, ST341), penicillin (0.1 g/L, Sigma-Aldrich, P3032), streptomycin (0.1 g/L, Sigma-Aldrich, S6501)) and transported to the laboratory within 2 h. A 2 mL volume of HEPES-199 (M&C GENE TECHNOLOGY LTD, CM10061) medium was aspirated using a syringe, and follicles were aspirated and sorted based on size to isolate COCs at the germinal vesicle (GV) stage in a 10 cm dish. Follicles were categorized as Small (< 3 mm; *n* = 40), Medium (3–5 mm; *n* = 34), and Large (> 5 mm; *n* = 35). The selected COCs were immediately examined under a stereo microscope (Nikon, SMZ745), washed, and transferred to 500 µL of PBS-PVA medium.

### 2.2 Collection of oocytes, cumulus granulosa cells, mural granulosa cells

Ovarian surface follicles were perforated with a hypodermic needle to aspirate cumulus-oocyte complexes (COCs) along with mural granulosa cells. Mural granulosa cells–which float freely in follicular fluid–exhibit a compact structure, dark cytoplasm, and flaky morphology. Cumulus granulosa cells form a tightly adherent layer surrounding the oocyte, displaying loose organization, clear cytoplasm, and a cloud-like appearance. During collection, mural granulosa cells were isolated from follicular fluid under stereomicroscopic guidance, while cumulus cells were obtained through standard COC denuding procedures. During the collection process, efforts were made to minimize the inclusion of red blood cells [23]. Following COC isolation, we used Tyrode’s solution (Sigma, T1788) to carefully remove the zona pellucida surrounding the oocytes. This ensured the recovery of contamination-free oocytes without cumulus granulosa cells. The removal of cumulus granulosa cells was confirmed via stereomicroscopic examination. Cumulus and mural granulosa cells were incubated in a medium containing hyaluronidase at 37 °C, followed by gentle pipetting or vortexing to degrade the hyaluronic acid matrix between cumulus cells. After dissociation, individual granulosa cells were carefully transferred into lysis buffer and then processed for subsequent steps, including library construction and high-throughput sequencing. During the experiment, the cumulus granulosa cells and mural granulosa cells were generally categorized and collected according to follicular size groups (small, medium, large).

### 2.3 Superovulation and in vivo matured MII oocyte collection

The synchronized ovulation protocol was employed using healthy adult sheep as oocyte donors. Animals were primed with progesterone using a controlled internal drug release (CIDR) device (EAZI-BREED® CIDR® Sheep and Goat Device; Pfizer Animal Health) containing 300 mg progesterone for 14 days. On Day 11, a series of follicle-stimulating hormone (FSH; Ningbo Hormone Products Co., Ltd.) injections were administered at 12-h intervals using the following descending dosage regimen: 75 IU, 75 IU, 60 IU, 60 IU, 45 IU, and 45 IU. Approximately 12 h after CIDR removal, donors received a single intramuscular injection of 350 IU pregnant mare serum gonadotropin (PMSG; Ningbo Hormone Products Co., Ltd.) to induce ovulation. All donors were simultaneously exposed to vasectomized rams for estrus detection. Food was withheld 12 h prior to surgery. At 48–60 h post-CIDR withdrawal, oocytes were aspirated from the oviducts via laparotomy. Ovulation status and corpus luteum formation were verified intraoperatively. A cannula-attached syringe needle was used to lavage oviducts with ∼20 mL flushing medium. The recovered medium was examined under a stereo microscope (Nikon SMZ745), and oocytes were selected based on morphological quality. Oocytes expelling the first polar body were identified as being in the metaphase II stage. These were treated with protease (Sigma, P8811) for zona pellucida removal, then transferred to single-cell lysis buffer. Processed oocytes underwent library preparation and subsequent high-throughput sequencing.

### 2.4 Single-cell cytoplasm-nucleus separation and in vitro methylation

Pick a single cell into the 2.5 µL lysis and run for 30 min at 37 °C, and then inactivate at 65 °C for 25 min. We added the lysate to a 3 µL nuclear-separation mixture, which contained 0.2 µL of Dynabeads MyOne Carboxylic Acid (65,011, Invitrogen), 5× First-Strand Buffer (18,064,071, Invitrogen), 10% Triton X-100 (T8787, Sigma-Aldrich), 40 U/µL RNase inhibitor, 20% Tween 20 (P1379, Sigma-Aldrich), and 100 mM dithiothreitol to isolate DNA and RNA. Then we vortexed and briefly centrifuged the mixture. Next, to attract the magnetic Dynabeads, we placed the tube that contained the mixture on a magnet, allowing us to separate the cytoplasm (RNA) in the supernatant from the Dynabeads-bound cell nucleus pellet (DNA).

### 2.5 Single-cell RNA library construction and sequencing

Firstly, the RNA-containing cytoplasm was transferred into 4.5 µL reverse transcription mix, which contained 200 U/µL SuperScript Ⅱ Reverse Transcriptase (18,064,071, Invitrogen), 10 µL template switch oligo primer, 40 U/µL RNase inhibitor, 10 µM 6-base pair (bp) barcode primer, 10 mM deoxynucleotide triphosphates (4030, Takara Bio Inc.), 5 M Betaine, and 1 M MgCl_2_ [17,20,21,24]. Next, we operated the polymerase chain reaction (PCR) at 25 °C for 5 min, 42 °C for 60 min, 50 °C for 30 min, and 70 °C for 10 min in a thermocycler on the mixture. Then, the purified cDNA was used in a biotin PCR and enriched. Next, we used the KAPA HyperPrep Kit (Illumina platforms) (KK8504, KAPA Biosystems Inc.) to construct the RNA libraries, which were sequenced with 150-bp paired-end reads on an Illumina HiSeq X Ten system (Illumina lnc.) [25].

### 2.6 Single-cell DNA library construction and sequencing

Selected the single oocytes with high-quality transcriptome data for DNA library preparation [[26], [27], [28]]. Firstly, the Dynabeads-bound cell nucleus (DNA) was treated with 5 µL protein lysis buffer, which consisted of 2.5 µL M-digestion buffer (D5044, Zymo Research), 0.5 µL proteinase K (P8107S, New England Biolabs) and 2 µL double-distilled H_2_O. Then, a single-cell whole-genome bisulfite sequencing protocol with minor modifications was used to construct the single-cell DNA libraries. Briefly, using an EZ-96 DNA Methylation-Direct™MagPrep (D5044, Zymo Research), we conducted the whole-genome bisulfite conversion. We performed four rounds of amplification to amplify the DNA strands efficiently, using Klenow (3′→5′ exo-) (NG202, ENzymics), 10 mM deoxynucleotide triphosphates, and scBS-seg-P5-N6-oligo1 (CTACACGACGCTCTTCCGATCTNNNNNN), with synthesis of the second strand followed, using scBS-seg-P7-N6-oligo2 (AGACGTGTGCTCTTCCGATCTNN NNN). Last, each DNA library was incorporated by the universal primers and index primers (New England Biolabs) and purified for each cell twice, with 0.8× AMPure XP beads (A63882, Beckman Coulter Life Sciences). To obtain 3 Gb of sequence with a 150-bp paired-end strategy, we sequenced the DNA library for each cell on an Illumina HiSeq X Ten system (IlluminaInc).

### 2.7 Single-cell transcriptomic data analysis

For single-cell transcriptomic data, we first separate the raw reads based on the barcode information added to the R2 end of paired-end reads during library preparation, enabling the distinction of individual cells. Subsequently, we compare the unique molecular identifier (UMI) information with the corresponding read1 data to eliminate template switch oligo (TSO) sequences and poly (A) tails. Next, we remove reads with low-quality bases (*n* > 10%) to obtain clean reads. The obtained clean reads are aligned to the sheep reference genome oviAri4 using TopHat (v2.1.1) [29]. Uniquely mapped reads are counted with HTSeq (v0.6.1) [30], disregarding reads with identical UMI sequences for each gene. Finally, the resulting count matrix is converted to transcripts per million (TPM).

Downstream analysis was performed using the Seurat (v4.0.0) package [31]. Data filtering criteria include expression of at least 1000 genes, mitochondrial read content below 40%, and a mapping ratio above 20%, retaining only data meeting all criteria for further analysis. Two thousand highly variable genes are selected for Manifold Approximation and Projection (UMAP) analysis. Pseudotime analysis is conducted using the Monocle2 (v2.22.0) package [32]. The CellChat (v1.5.0) package [33] is applied to analyze cell-cell communication. Differences between cell types are assessed with the Wilcoxon rank-sum test to identify differentially expressed genes (DEGs), and *P*-values are adjusted using the Benjamini-Hochberg (BH) method. DEGs are defined as genes with an adjusted *P*-value ≤ 0.05 and |log2FoldChange| ≥ 0.5.

Gene Ontology (GO) and Kyoto Encyclopedia of Genes and Genomes (KEGG) analyses are performed using DAVID [34]. Additionally, gene set enrichment analysis (GSEA) is conducted using the ClusterProfiler (v4.8.1) package [35].

### 2.8 DNA data processing

For DNA data, Trim Galore (v0.6.6) is first utilized in paired-end mode to remove random primers, eliminate low-quality bases, and trim adapters from single-cell DNA data. The length of each trimmed read is ensured to be greater than 50 base pairs. Subsequently, the trimmed clean reads are aligned to the sheep reference genome oviAri4 in paired-end mode using Bismark Bisulfite Mapper (v0.7.6) [36]. For reads that fail to align in paired-end mode, single-end mode is employed for alignment to oviAri4. PCR duplicates are removed using SAMtools (v0.1.18) [37]. Finally, for the aligned data, it is ensured that each cell in the DNA library covers > 500,000 WCG (ACG/TCG) sites and 5 million GCH (GCA/GCC/GCT) sites, with a bisulfite conversion rate ≥ 94% and a genome coverage ≥ 3%.

### 2.9 Quantitative analysis of WCG and GCH methylation levels

For quantifying DNA methylation levels, sequencing data with at least 1× coverage are used for downstream analysis. GCG sites are excluded due to challenges in distinguishing DNA methylation from chromatin accessibility data. Similarly, CCG sites are excluded due to the low activity of M.CviPI methyltransferase at CC sites.

### 2.10 Definition of nucleosome-depleted regions

A 100 bp sliding window with a 20 bp step size is used to calculate GCH methylation levels within each window. Windows with GCH methylation levels significantly above the genomic background (*P*-value ≤ 10−20, determined by Chi-square test), spanning > 140 bp, and covering at least 5 GCH sites are identified as nucleosome-depleted regions (NDRs). Based on their positions relative to transcription start sites (TSS), NDRs are classified as proximal or distal. Proximal NDRs are defined as regions within 2 kb upstream or downstream of the TSS, while distal NDRs are located > 2 kb away from the TSS.

### 2.11 Identification of differential NDRs and differentially methylated regions (DMRs)

Following previous studies, differential NDRs are used to compare chromatin accessibility between different cell types. For identified NDRs, the Wilcoxon rank-sum test is applied to compare NDRs across cell types. In each cell type, NDRs with GCH methylation levels higher than those of other groups by > 0.1 and a *P*-value ≤ 0.05 are defined as upregulated (more open). Conversely, NDRs with GCH methylation levels lower than those of other groups by > 0.1 and a *P*-value ≤ 0.05 are defined as downregulated (more closed).

For the identification of DMRs, we proceed as follows. The entire genome is divided into 500 bp bins, and the average methylation level for each bin is calculated. A DMR is defined as upregulated if the methylation level in this cell type is greater than 0.2 compared to other cell types, with a *P*-value ≤ 0.05 determined by the Wilcoxon rank-sum test. Conversely, a DMR is defined as downregulated if the methylation level in this cell type is < 0.2 compared to other cell types, also with a *P*-value ≤ 0.05 determined by the Wilcoxon rank-sum test.

### 2.12 Motif enrichment analysis

We used findMotifsGenome.pl in HOMER (v4.11.1) [38] with parameters “-size given -size 2000 -len 8 -S 100” to analyze motif enrichment in the proximal and distal NDRs of Type 1, Type 2, and Type 3 oocytes.

### 2.13 Enrichment analysis of NDRs in genomic regions and repetitive elements

For different genomic regions and repetitive elements, corresponding positions in oviAri4 were obtained from UCSC. We define the region from 2 kb upstream to 2 kb downstream of the TSS as the promoter region. The region from the TSS to the transcription end site (TES) is defined as the gene body region. We calculated the frequency of all NDRs within these genomic regions and repetitive elements for each cell type. Additionally, the genome was randomly divided into bins at a rate of 100 times the number of NDRs, and the frequency of these bins falling within genomic regions and repetitive elements was calculated to serve as the background. The enrichment score was determined by comparing the frequency of NDRs with the background frequency.

### 2.14 RNA quantification and real-time PCR

With the amplification conditions of 40 cycles with initial denaturation at 95 °C for 30 s followed by denaturation at 95 °C for 5 s and annealing at 60 °C for 30 s, we used the Q225 Sequence Detection System to perform Real-time PCR. Using the 2-ΔΔct method with *GAPDH* as the reference gene, we calculated the relative gene expression. Then the primer sequences were listed in Table S4.

### 2.15 In vitro maturation, fertilization and embryo culture

The composition of the IVM medium used in this study was as follows: 8 mL M199 (Gibco, 11,150,059), 1.5 mL FBS (Gibco, 30,044,333), 500 µL penicillin-streptomycin (Gibco, 15,140), 0.23 mM sodium pyruvate (Sigma, P4562), 2 mM L-glutamine (Sigma, G3126), 5 µg/mL follicle-stimulating hormone (FSH, Sigma, 869,001), 5 µg/mL luteinizing hormone (LH, Sigma, 869,003), 10 ng/mL epidermal growth factor (EGF, Sigma, E9644), and 2 µg/mL estradiol (E2, Sigma, E8875). Evaluated the COCs and statistically analyzed the cumulus expansion under the Stereo Microscopes after 22 h in vitro maturation. Transferred the oocytes into hyaluronidase (1% w/v) and completely removed the cumulus cells by pipetting using a glass capillary during washing, and then selected the polar bodies from cumulus-free oocytes under a Stereo Microscope using a glass capillary. Incubated the matured oocytes with capacitated sperm at a concentration of 1 × 10^−5^/mL in 500 µL IVF medium for 20 h at 38.5 °C, 5% CO_2_, then saturated the humidity. Next, transfer the zygotes into in vitro culture (IVC) medium. Measured the cleavage rate 48 h later.

### 2.16 ET-1 and BQ123 treatment in the process of IVM and IVC

To screen for the optimal working concentration, ET-1 (Sigma, 05-23-3800) and BQ123 (MCE, HY-12378) were supplemented in the IVM medium at concentrations of 1 nM, 10 nM, and 100 nM, respectively. Based on the rate of first polar body extrusion, 10 nM of ET-1 and BQ123 were selected for subsequent experiments. After 168 h of IVC, the blastocyst rates were assessed.

### 2.17 Detecting ROS levels and GSH levels in MII oocytes

In subsequent experiments, such as ROS and GSH, the concentrations of ET-1 and BQ123 used were both 10 nM. Incubated a total number of 300 mature oocytes (three replicates) with 10 µM oxidation-sensitive fluorescent probe DCFH (Reactive Oxygen Species Assay Kit, Beyotime, S0033S) and 10 µM of Cell Tracker Blue (Thermo Fisher Scientific, Barcelona, Spain; C12881) in PBS-PVA medium for 30 min at 38.5 °C, 5% CO_2_ and then saturated the humidity to detect intracellular reactive oxygen species (ROS) and glutathione (GSH). Then the samples were washed three times with PBS-PVA medium. We used laser Stereo Microscopes to examine the samples and further evaluate them using ImageJ 1.45s software (National Institutes of Health, Bethesda, USA). Mito-Tracker, ER-Tracker, and Golgi-Tracker Red Staining Incubated a total of 450 mature oocytes (three replicates) in PBS-PVA medium for 30 min at 38.5 °C, 5% CO_2_, with 200 nM Mito-Tracker Red (Mito-Tracker Red CMXRos, Beyotime, C1035), ER-Tracker Green (ER-Tracker Green, Beyotime, C1042S, 1:1000), and Golgi-Tracker Red (Golgi-Tracker Red, Beyotime, C1043), then saturated the humidity. The oocytes were washed three times in PBS-PVA medium and placed on slides for evaluation. Then, the oocytes were counterstained with DAPI (10 µg/mL) at room temperature for 10 min. The oocytes were washed three times in PBS-PVA (5 min per wash with agitation) and observed via epifluorescence microscopy. The fluorescence intensity was subsequently evaluated using ImageJ 1.45s software. (National Institutes of Health, Bethesda, USA).

### 2.18 Analysis of cytoplasmic organelles in oocytes treated with ET-1 and BQ123

The distribution of cytoplasmic organelles including mitochondria, endoplasmic reticulum (ER), Golgi apparatus and cortical granules (CGs), was assessed. For mitochondria, ER, and Golgi apparatus analysis, a total of 450 oocytes (across three replicates) were incubated in PBS-PVA medium at 38.5 °C and 5% CO₂ for 30 min with the following organelle-specific kits: 200 nM MitoTracker Red CMXRos (Beyotime, C1035), ER-Tracker Green (Beyotime, C1042S, diluted 1:1000), and Golgi-Tracker Red (Beyotime, C1043). Oocytes were then washed three times in PBS-PVA medium and mounted on slides. Nuclei were counterstained with DAPI (10 µg/mL) at room temperature for 10 min, followed by three 5 min agitation washes in PBS-PVA. Fluorescence intensity was observed using epifluorescence microscopy and quantified using ImageJ 1.45s software (National Institutes of Health, Bethesda, USA).

For CGs analysis, a separate set of 450 oocytes (three replicates) was fixed in 4% paraformaldehyde (PFA) for 30 min and blocked with 0.3% BSA solution. After three 5 min agitation washes in PBS-PVA, oocytes were permeabilized in 1% Triton X-100 (T8787) for 1 h at room temperature. CGs were stained by incubating oocytes in 30 µL droplets of 100 µg/mL fluorescein isothiocyanate-conjugated peanut agglutinin (FITC-PNA; Thermo Fisher Scientific, L21409) for 30 min at 37 °C on a heated plate in the dark. Fluorescence imaging was performed using an epifluorescence microscope (Nikon Ti2) equipped with appropriate filter sets. Quantitative analysis of fluorescence intensity and spatial distribution patterns was conducted using ImageJ 1.45s software.

Organelle distribution patterns were systematically classified into two categories: “normal”, defined by homogeneous cytoplasmic dispersion, or “abnormal”, characterized by clustered granulations, asymmetric cortical aggregation, or cavitation anomalies. Each experimental group consisted of > 50 oocytes, with three biological replicates conducted. The normal distribution rate was calculated for each group as the percentage of oocytes exhibiting normal distribution, defined as: (Number of oocytes with normal distribution/Total number of oocytes analyzed) × 100%. Statistical analysis was then performed using one-way analysis of variance (ANOVA) [[39], [40], [41], [42], [43]].

### 2.19 Mitochondrial membrane potential (ΔΨm) measurement and evaluation of total ATP content

Oocyte mitochondrial membrane potential was assessed with the Mito-Probe JC-1 Assay Kit (Beyotime, C2003S). Following incubation with 2 µM JC-1 in PBS-PVA medium at 38.5 °C, 5% CO₂, and saturated humidity, the oocytes were washed three times in PBS-PVA medium and subsequently analyzed by epifluorescence microscopy. JC-1 dye was equipped with a fluorescence emission of green (529 nM) and red (590 nM). Thus, the red/green fluorescence intensity ratio was measured to indicate Mitochondrial membrane potential (ΔΨm).

The ATP content of mature oocytes was measured using an ATP Bioluminescence Assay Kit (Beyotime, S0027) according to the manufacturer’s instructions. A total of 450 oocytes were used in three independent replicates. For each measurement, 25 oocytes were lysed in 50 µL of ATP lysis buffer on ice, vortexed for 7 min, and then centrifuged at 12,000 × g for 5 min at 4 °C. The resulting supernatant was subsequently transferred to a 96-well black culture plate for analysis. Then, read the samples and standards with a luminometer (Synergy4, Bio-Tek). Finally, the ATP level was calculated according to the standard curve.

### 2.20 Immunofluorescent staining

Following the removal of the zona pellucida with 0.5% pronase, a total of 79 denuded mature oocytes (in four replicates) were fixed in 4% paraformaldehyde for 15 min at room temperature and then permeabilized with 0.5% Triton X-100 for 1 h. Then the oocytes were blocked with 2% BSA in PBS for 6 h at room temperature. Incubated the oocytes/blastocyst with primary antibodies (α-tubulin Polyclonal antibody, 1:200, Proteintech, 11224-1-AP; CDX2 Polyclonal antibody, 1:500, BioGenex, MU392A-5UC; OCT4 Polyclonal antibody, 1:500, Proteintech, 11263-1-ap) at 4 °C overnight. After being washed three times under a 5 min agitation in PBS-PVA, the oocytes were probed with Alexa Fluor 488 goat anti-rabbit IgG (1:200, Thermo Fisher Scientific, A21206) for 1 h at 38.5 °C. Counterstained the oocytes with DAPI (10 µg/mL) at room temperature for 10 min. And then, the oocytes were washed extensively with PBS-PVA medium, evaluated using a Nikon A1 confocal microscope (Nikon Instruments Europe, Amsterdam, The Netherlands) and ImageJ 1.45s software (National Institutes of Health, Bethesda, USA). Used the lasers of 408 and 488 nM to excite Hoechst and dyes sequentially.

Fixed the oocytes in 4% paraformaldehyde in PBS for 15 min at room temperature to identify the distribution pattern of cortical granules. Permeabilized the oocytes in 1% Triton for 1 h at room temperature and stained them in 100 µg/mL of fluorescein isothiocyanate-conjugated peanut agglutinin (FITC-PNA; Invitrogen©, Thermo Fisher Scientific, Barcelona, Spain; L21409) for 30 min at 38.5 °C. Counterstained the oocytes with DAPI (10 µg/mL) at room temperature for 10 min. Then the oocytes were washed extensively with PBS-PVA medium, observed the fluorescence intensity by Nikon A1 confocal microscope (Nikon Instruments Europe, Amsterdam, The Netherlands), and further evaluated using ImageJ 1.45s software (National Institutes of Health, Bethesda, USA).

### 2.21 Data availability

The raw sequence data reported in this paper have been deposited in the Genome Sequence Archive [44] (Genomics, Proteomics & Bioinformatics 2021) in National Genomics Data Center [45], China National Center for Bioinformation/Beijing Institute of Genomics, Chinese Academy of Sciences (GSA: CRA020425) which are publicly accessible at https://ngdc.cncb.ac.cn/gsa.

## 3 Results and discussion

### 3.1 Single-cell multi-omics analysis during the maturation process of antral follicles in sheep

**Fig. 1:**
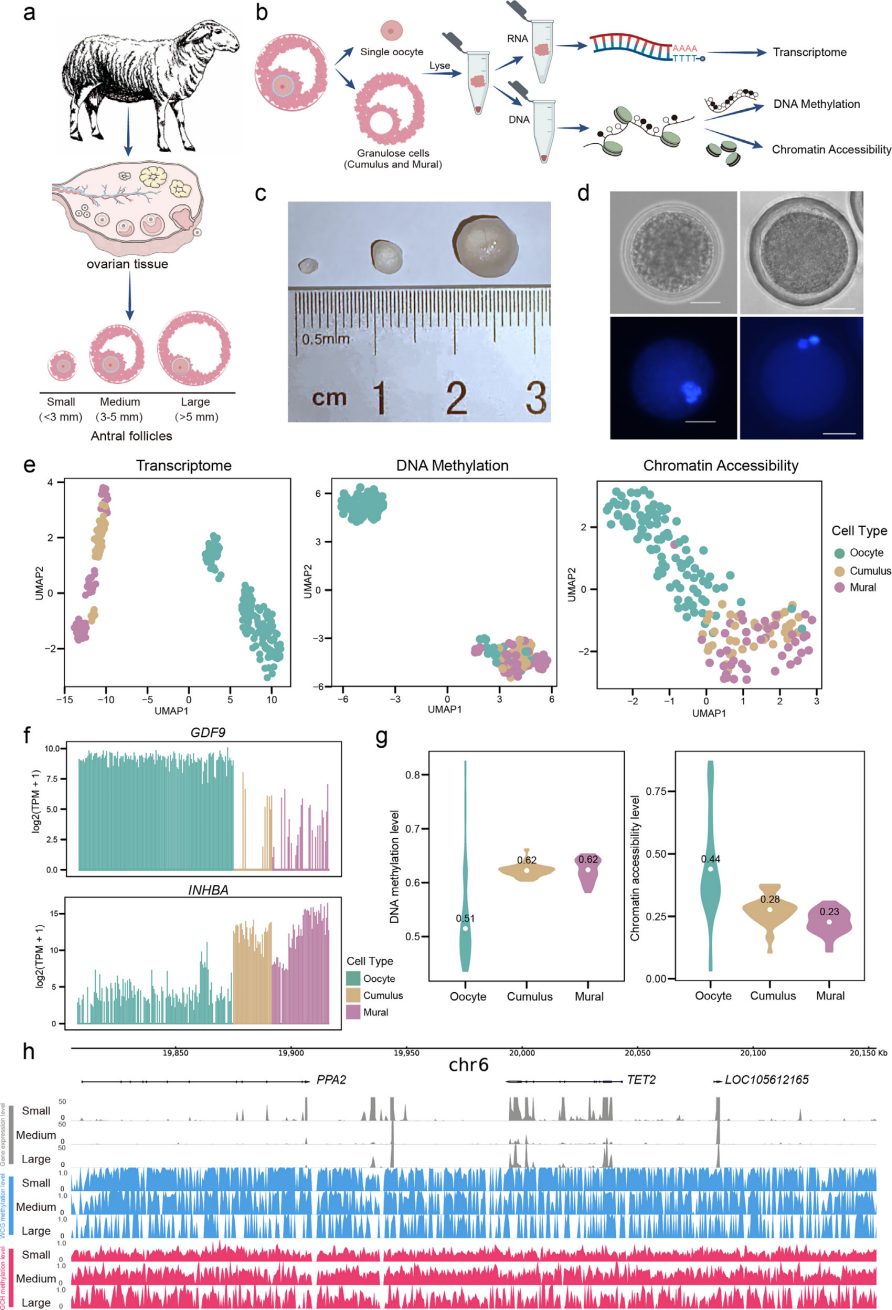
**Single-cell multi-omics analysis of sheep oocytes and granulosa cells.** (a,b) Schematic representation of the collection and processing of sheep oocytes and granulosa cells for single-cell multi-omics sequencing analysis. (c) Representative images classified by follicle size from left to right (small: < 3 mm, medium: 3–5 mm, large: > 5 mm). (d) Hoechst staining determines the oocyte stages. Left: Representative bright-field and Hoechst-stained images of GV stage oocytes; Right: Representative bright-field and Hoechst-stained images of MII stage oocytes. Scale bar, 50 µm. (e) UMAP analysis reveals the clustering patterns of transcriptomics, DNA methylation (WCG), and chromatin accessibility (GCH) in oocytes and granulosa cells. (f) Expression profiles of oocyte marker (*GDF9*) and granulosa cell marker (*INHBA*) in oocytes, cumulus granulosa cells, and mural granulosa cells. (g) DNA methylation and chromatin accessibility status of oocytes, cumulus granulosa cells, and mural granulosa cells, with white lines representing the average levels for each cell type. (h) Visualization of gene expression, DNA methylation patterns, and chromatin accessibility in oocytes from follicles of different sizes.

Antral follicles with normal morphology were collected from sheep ovaries and then high-quality COCs and mural granulosa cell samples were obtained. Subsequently, oocytes and cumulus granulosa cell samples were isolated from COCs. Due to the low abundance of RNA in single granulosa cell, each cumulus granulosa cell sample and mural granulosa cell sample consisted of 10 randomly selected granulosa cells from the same antral follicle [13], respectively (Fig. 1a–c). According to follicle size, the collected samples were categorized into three types: Small (< 3 mm; *n* = 40), Medium (3–5 mm; *n* = 34), and Large (> 5 mm; *n* = 35). Simultaneously, to analyze and depict the developmental fate of oocytes in antral follicles, we also collected MII-stage oocytes (*n* = 28) and their corresponding cumulus granulosa cell samples. The Hoechst staining of the collected oocytes from antral follicles confirmed their presence in the GV stage, with intact cellular structures and well-maintained morphology (Fig. 1d). Subsequently, using our modified single-cell multi-omics technology, cytoplasm and nuclei were separated from single oocyte and each granulosa cell sample to construct RNA and DNA libraries separately. Transcriptome sequencing was performed using modified single-cell tag reverse transcription sequencing (STRT-seq) method [24], and DNA methylation and chromatin accessibility were assessed using improved single-cell chromatin overall omic-scale sequencing (scCOOL-seq) technology. DNA methylation status was inferred based on endogenous methylation at WCG sites, and chromatin accessibility was inferred based on in vitro methylation at GCH sites [14,15,20]. Utilizing the two methods above, we achieved a tri-omics analysis for each oocyte and granulosa cell sample.

Following rigorous quality control screening of the obtained RNA and DNA data, a total of 135 oocyte samples, 35 cumulus granulosa cell samples, and 52 mural granulosa cell samples were retained for subsequent analysis. Among them, each cell sample detected an average of 8679 genes and 655,351 UMIs. Meanwhile, an average of 2,794,599 WCG sites and 19,271,540 GCH sites were covered for each cell sample (Fig. S1a; Table S1). The Pearson correlation heatmap among different datasets indicates good data quality (Fig. S1b). Visualization of the sequencing data using UMAP method revealed that the three omics data for all cells were grouped into two main clusters, corresponding to oocytes and granulosa cells (including cumulus granulosa cells and mural granulosa cells) (Fig. 1e). Based on the expression levels of the reported oocyte marker gene Growth Differentiation Factor 9 (*GDF9*) [46] and granulosa cell marker gene Inhibin Subunit Beta A (*INHBA*) [47] in our transcriptomic data, it revealed differential expression levels of these two markers in oocytes and granulosa cells (Fig. 1f). Analysis of the DNA methylation levels and chromatin accessibility in oocytes and granulosa cells indicated that, compared to cumulus granulosa cells (DNA methylation level: 0.62, chromatin accessibility: 0.28) and mural granulosa cells (DNA methylation level: 0.62, chromatin accessibility: 0.23), oocytes exhibited a lower average DNA methylation level (0.51) and higher chromatin accessibility (0.44). Furthermore, oocytes displayed greater heterogeneity in both DNA methylation levels and chromatin accessibility (ranging from 0.44 to 0.82 and 0.03 to 0.87, respectively) (Fig. 1g). In conclusion, using our modified single-cell multi-omics sequencing technology, we obtained high-quality datasets for the transcriptome, DNA methylation, and chromatin accessibility of oocytes and granulosa cells from ovine antral follicles with different diameters (Fig. 1h).

### 3.2 Transcriptional features of oocytes during the development of antral follicles in sheep

**Fig. 2:**
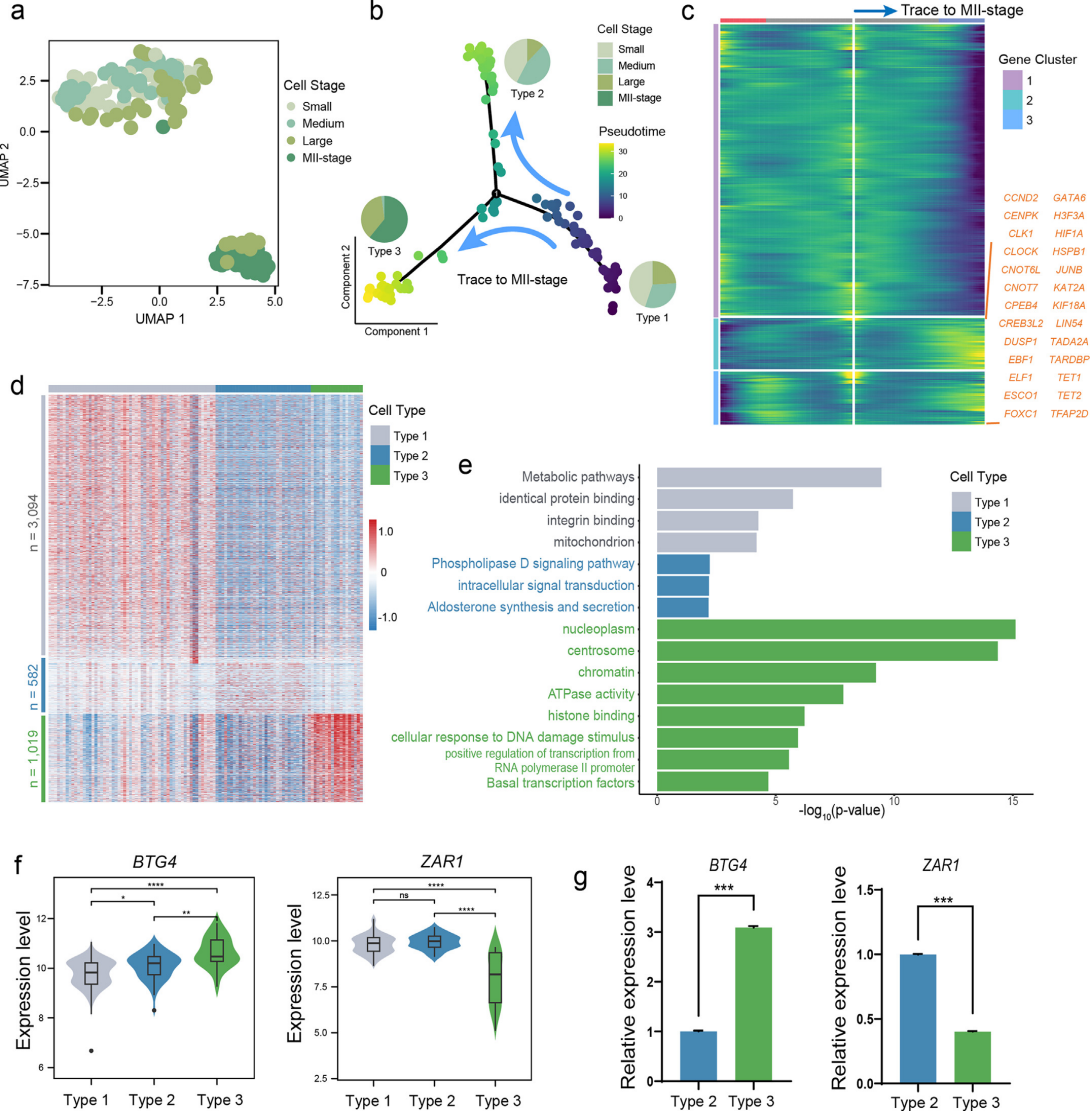
**Transcriptional changes associated with different maturation fates of sheep oocytes.** (a) UMAP dimensionality reduction analysis of oocytes from follicles of different sizes and MII stage oocytes. (b) Pseudotime trajectory distribution of oocytes from follicles of different sizes and MII stage oocytes. The pie chart shows the proportion of oocytes from follicles of various sizes and MII stage oocytes across different trajectory branches (Types). (c) Heatmap of gene dynamics along two maturation fate trajectories. The blue section at the top of the heatmap represents the direction towards the Type 3 branch (which includes MII-stage oocytes), while the red section represents the direction towards the Type 2 branch. (d) Heatmap of differentially expressed genes among the three types of GV stage oocytes. (e) GO enrichment analysis of upregulated DEGs in Type 1, Type 2, and Type 3. (f) Expression differences of important genes during oocyte maturation among the three Types. *P < 0.05, **P < 0.01, ****P < 0.0001. (g) Real-time PCR validation of important genes involved in oocyte maturation in Type 2 and Type 3 oocytes. *n* = 3. ***P < 0.001.

To better investigate the dynamic changes in oocytes during the development of antral follicles in sheep, we interrogated a single-cell RNA-seq atlas covering all follicular stages. All oocytes were successfully clustered into two groups: one comprising oocytes harvested from large antral follicles and MII-stage oocytes forming one cluster, while a second containing the remaining oocytes (Fig. 2a). Next, through the analysis of pseudo-temporal simulation of cell development trajectories, we identified two developmental branches for all oocytes. Notably, one branch ultimately led to MII-stage oocytes, which contained the majority originating from oocytes of large antral follicles. Therefore, based on the results of the pseudo-temporal simulation of cell developmental trajectories, we redefined all oocyte types as Type 1, Type 2, and Type 3. These three types of oocytes exhibited relatively uniform expression of oocyte marker genes (Fig. S1c) and corresponded to an earlier stage of antral follicular development, a developmental branch divergent from maturation, and a developmental branch toward maturation, respectively (Figs. 2b and S2a,b). Subsequently, we analyzed the dynamic gene changes in the two developmental branches. We observed a significant downregulation of many genes in oocytes within the developmental fate towards the MII stage. This finding aligns with previous research results on genome-wide transcriptional inactivation during follicular development and oocyte maturation [48,49]. Additionally, we identified key genes such as cyclin D2 (*CCND2*), crucial for the G1/S transition in the cell cycle and highly significant during follicular development [50,51]; centromere protein K (*CENPK*), essential for vertebrate centromere assembly and chromosome segregation [52]; and GATA binding protein 6 (*GATA6*), a critical transcriptional regulatory factor, playing a key role in ovarian follicle development [53], gradually upregulated during the developmental fate towards MII-stage (Fig. 2c).

To facilitate a more in-depth comparison among oocytes from antral follicles with distinct developmental fate, we employed the redefined categories of Type 1, Type 2, and Type 3 for subsequent analyses. To prevent potential differential outcomes due to the distinct stages of MII-stage and GV-stage in oocytes, we excluded MII-stage oocytes from the Type 3 category in subsequent analyses. We analyzed the DEGs among the three types of oocytes (Fig. 2d; Table S2). Consistent with the findings from the analysis of the dynamic gene changes (Fig. 2c), Type 3 oocytes exhibited a significant downregulation of numerous genes. Simultaneously, we conducted GO enrichment analysis on the upregulated genes in each type of oocyte. The results indicate that the DEGs from Type 1 oocytes are significantly enriched in metabolic pathway biological processes and mitochondrial cellular components, which are associated with the more active biological processes exhibited by earlier-stage oocytes [54,55] (Fig. 2e). The Phospholipase D signaling pathway, intracellular signal transduction, and Aldosterone synthesis and secretion were enriched in Type 2 oocytes. Additionally, cellular components such as nucleoplasm, centrosomes, chromatin, and biological processes including ATPase activity, histone binding, cellular response to DNA damage stimulus, and positive regulation of transcription from RNA polymerase II promoter were enriched in Type 3 oocytes (Fig. 2e). These findings align with the developmental fate of oocytes at different stages. Compared to the earlier-stage Type 1 oocytes, Type 2 oocytes may be destined for atresia, while Type 3 oocytes appear to be on the verge of maturation. Additionally, we observed an upregulation of B-cell translocation gene-4 (*BTG4*) in Type 3, a gene that mediates crucial steps in cytoplasmic maturation of oocytes [56]; downregulation of Zygote arrest-1 (*ZAR1*), a gene responsible for maintaining the transcriptome stability of oocytes and regulating mRNA translation activation [57] (Fig. 2f). The expression patterns of these genes can be validated in oocytes with two distinct developmental fates using quantitative PCR methods (Fig. 2g). The above results demonstrate distinct gene expression patterns of oocytes during the development of antral follicles.

### 3.3 Epigenetic features of oocytes during the development of ovine antral follicles

**Fig. 3:**
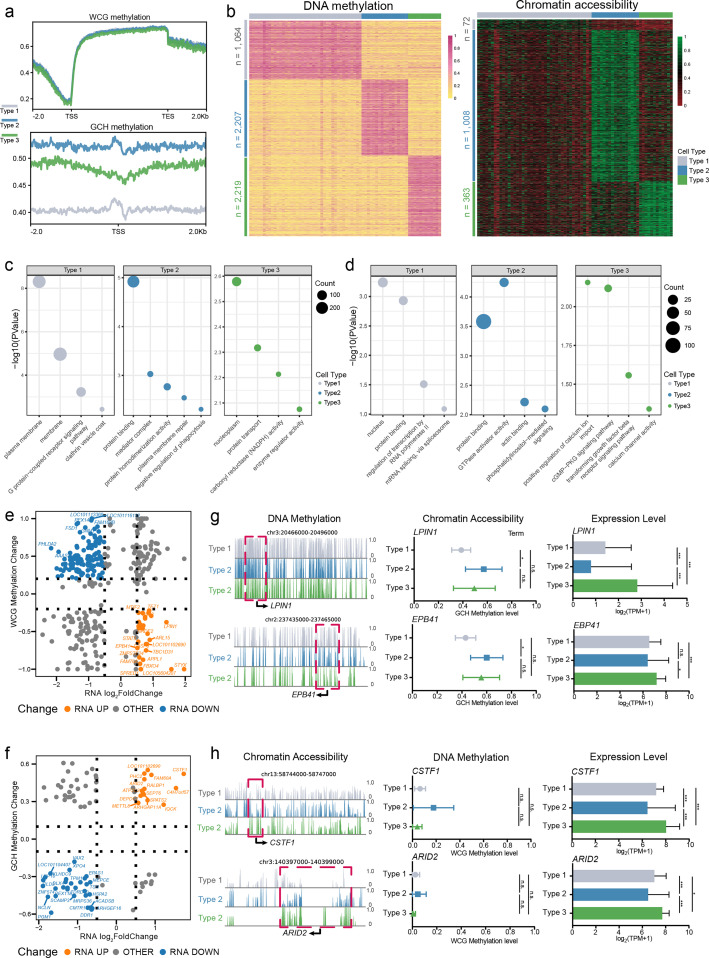
**Epigenetic changes and integrative analysis of different maturation fates in sheep oocytes.** (a) Global levels of DNA methylation and chromatin accessibility in the three types of oocytes. Top: DNA methylation distribution across the gene body region, including the 2 kb regions upstream and downstream of the TSS and TES, for the three types of oocytes. Bottom: Chromatin accessibility status within 2 kb upstream and downstream of the TSS in the three types of oocytes. (b) Heatmap of DMRs and differential NDRs in the three types of oocytes. (c) GO enrichment analysis of upregulated DMRs in the promoter regions of Type 1, Type 2, and Type 3 oocytes. (d) GO enrichment analysis of upregulated differential NDRs in the promoter regions of Type 1, Type 2, and Type 3 oocytes. (e) Integrative analysis of DEGs and promoter region DMRs in Type 3 oocytes. (f) Integrative analysis of DEGs and differential NDRs in promoter regions of Type 3 oocytes. (g) Schematic representation of DNA methylation sites for *LPIN1* (top) and *EPB41* (bottom) across the genome. Left: Dashed boxes indicate representative DMRs. Middle: Chromatin accessibility levels in the promoter regions of the three types of oocytes, with dots representing mean values. Right: Bar charts showing average gene expression levels for the three types of oocytes. (h) Schematic representation of chromatin accessibility sites for *CSTF1* (top) and *ARID2* (bottom) across the genome. Left: Dashed boxes indicate representative NDRs. Middle: DNA methylation levels in promoter regions of the three types of oocytes, with dots representing mean values. Right: Bar charts showing average gene expression levels for the three types of oocytes. *P < 0.05, ***P < 0.001.

Following, we conducted an in-depth analysis of DNA methylation and chromatin accessibility of oocytes during the development of antral follicles. Initially, we observed a relatively similar distribution pattern of DNA methylation in the gene body regions, from the TSS to the TES, among the three types of oocytes (Fig. 3a). Then, we utilized the GCH methylation level to assess chromatin accessibility. We found distinct characteristics in chromatin accessibility among the three types of oocytes: Type 1 oocytes exhibited a relatively lower open state in the TSS region, while Type 2 oocytes were in the highest open state, and Type 3 oocytes were positioned between the two (Fig. 3a). We also quantitatively assessed the global levels of DNA methylation and chromatin accessibility across the three oocyte types (Fig. S3a,b). At the global level, differences among these oocytes were relatively modest.

To further investigate the impact of DNA methylation and chromatin accessibility during the growth of antral follicles, Differentially Methylated Regions (DMRs) and Differential Nucleosome-Depleted Regions (NDRs) were then analyzed in the three types of oocytes (Fig. 3b). The GO enrichment analyses were performed on the hypermethylated DMRs and NDRs within the promoter regions of these three types of oocytes. The results indicate that for the hypermethylated DMRs in the promoter regions, Type 1 oocytes are enriched in processes related to the plasma membrane, membrane, G protein−coupled receptor signaling pathway, and clathrin vesicle coat. Type 2 oocytes are enriched in processes involving protein binding, mediator complex, protein homodimerization activity, plasma membrane repair, and negative regulation of phagocytosis. Type 3 oocytes show enrichment in processes associated with nucleoplasm, protein transport, carbonyl reductase (NADPH) activity, and enzyme regulator activity (Fig. 3c). For the hypermethylated NDRs in the promoter regions, Type 1 oocytes are enriched in processes associated with the nucleus, protein binding, regulation of transcription by RNA polymerase II, and mRNA splicing via the spliceosome. Type 2 oocytes show enrichment in processes involving protein binding, GTPase activator activity, actin binding, and phosphatidylinositol−mediated signaling. Type 3 oocytes are enriched in processes related to the positive regulation of calcium ion import, the cGMP−PKG signaling pathway, transforming growth factor beta receptor signaling pathway, and calcium channel activity (Fig. 3d). These enrichment results are generally consistent with the corresponding states of the three types of oocytes.

Subsequently, the enrichment patterns of NDRs in different genomic elements for each type of oocyte were investigated. Furthermore, compared with MII-stage, the enrichment of epigenetic elements in Type 3 oocytes from large antral follicles exhibit a similar enrichment pattern to MII-stage oocytes, while Type 2 oocytes show a distinct pattern. This reflects the differential enrichment patterns of genomic elements in oocytes from the two developmental branches (Fig. S3c). Finally, given the relatively limited enrichment observed in proximal NDRs, with only a few motifs identified in each oocyte type and modest enrichment levels, we focused our analysis on the transcription factor motif enrichment within distal NDRs across the three oocyte types (Fig. S3d–g). Notably, the Type 3 oocytes exhibited significant enrichment of key transcription factors, including *CTCF, POU5F1* (*OCT4*), and *STIL*, which are closely associated with transcriptional regulation, cell pluripotency, and cell cycle control [[58], [59], [60], [61]] (Fig. S3d).

### 3.4 The interplay between epigenetic modifications and gene expression during the maturation of antral follicles

To further elucidate the association between epigenetic modification and gene expression during the maturation of sheep oocytes from antral follicles, the DEGs in Type 3 with DMRs and Differential NDRs were integrated. We conducted separate correlation analyses for the DNA methylation-regulated transcriptome and the chromatin accessibility-regulated transcriptome, revealing a multitude of genes associated with both epigenetic regulations (Fig. 3e,f). For instance, lipin 1 (*LPIN1*), a gene highly associated with phosphatidic acid metabolism and mitochondrial fission [62]; erythrocyte membrane protein band 4.1 (*EPB41*), a gene essential for the assembly of the cell cytoskeleton and recruitment of the protein-protein complex in the post-mitotic phase of cell cortex [63], with their expression significantly correlated with DNA promoter region methylation regulation (Fig. 3g). Furthermore, cleavage stimulation factor subunit 1 (*CSTF1*), a critical gene encoding one of the three subunits forming the cleavage stimulation factor (*CSTF*), is involved in polyadenylation of precursor mRNA and 3′ end cleavage [64]; AT-rich interactive domain-containing protein 2 (*ARID2*), a gene highly associated with lineage-specific gene regulation, cell cycle control, transcriptional regulation, and chromatin structure modification [65], whose expression is significantly correlated with the chromatin accessibility of gene promoter regions (Fig. 3h). The genes mentioned above may serve as valuable references for future in-depth studies on the epigenetic regulatory mechanisms underlying the maturation process of sheep oocytes (Table S3).

### 3.5 Differential analysis of oocytes along two developmental fate trajectories

**Fig. 4:**
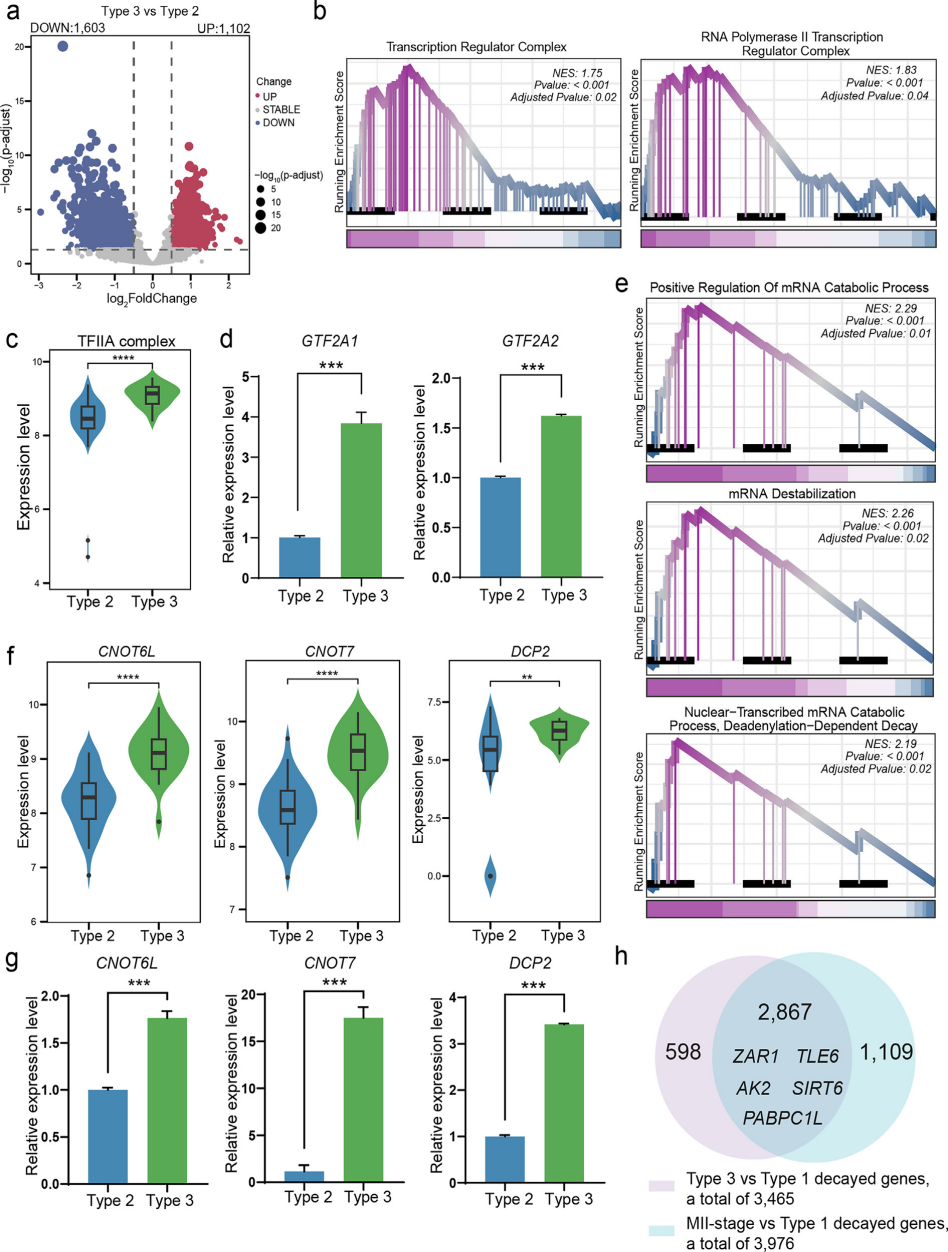
**Differential analysis between Type 3 and Type 2 oocytes.** (a) Volcano plot showing DEGs identified in the comparison between Type 3 and Type 2 oocytes. (b) GSEA enrichment of genes upregulated in Type 3 compared to Type 2 oocytes in processes related to the transcription regulator complex. (c) Expression differences of transcription factor complex TFIIA between Type 2 and Type 3 oocytes. ****P < 0.0001. (d) Real-time PCR validation of the two genes forming the transcription factor complex TFIIA in Type 3 and Type 2 oocytes. *n* = 3. ***P < 0.001. (e) GSEA enrichment of genes upregulated in Type 3 compared to Type 2 oocytes in mRNA degradation-related processes. (f) Expression differences of three key genes involved in maternal mRNA degradation in Type 2 and Type 3 oocytes. **P < 0.01, ****P < 0.0001. (g) Real-time PCR validation of three key genes involved in maternal mRNA degradation in Type 2 and Type 3 oocytes. *n* = 3. ***P < 0.001. (h) Combined analysis of genes degraded in Type 3 oocytes compared to Type 1 and in MII oocytes compared to Type 1.

In the aforementioned multi-omics analysis, Type 3 and Type 2 oocytes exhibited two distinct developmental fates. Type 3 oocytes had the potential capacity to develop to MII oocytes, whereas Type 2 oocytes did not. Therefore, to specifically investigate the differences in oocytes with varying developmental fate trends, we conducted a comparative analysis of Type 3 and Type 2 oocytes. Compared with Type 2 oocytes, the Type 3 oocytes exhibited 1102 upregulated genes and 1603 downregulated genes (Fig. 4a). We performed GSEA on all genes generated from the differential expression analysis results. The results indicated that Type 3 exhibited enrichment in two upregulated cellular components: Transcription Regulator Complex and RNA Polymerase II Transcription Regulator Complex (Fig. 4b). We also observed distinct expression patterns of five general transcription factor complexes (including core and holo forms of the TFIIH complex) that constitute the above two cellular components in the two types of oocytes (Figs. 4c and S4a). It is noteworthy that the TFIIA complex is significantly downregulated in Type 2 compared to Type 3 (Fig. 4c). Previous studies suggest that the TFIIA complex plays a crucial role in the degradation of mRNA during the maturation of the oocyte transcriptome [66]. Therefore, we speculate that this general transcription factor may have a certain influence on driving oocytes toward the two developmental branches. Verification of the gene expression of the two subunits constituting the TFIIA complex is conducted using quantitative PCR, and the results are consistent with our sequencing observations (Fig. 4d).

Interestingly, GSEA results also revealed an upregulation of biological processes associated with mRNA degradation in Type 3 (Fig. 4e), aligning with the described functions of the general transcription factor TFIIA mentioned earlier. During development, the degradation of maternal RNA occurs in two distinct types, namely maternal (M)-decay and zygotic (Z)-decay [67,68]. It is generally believed that human M-decay initiates gradually during the GV stage and is mostly completed by the MII stage [68]. Furthermore, we observed a significant upregulation of commonly reported factors involved in M-decay, such as *CNOT6L, CNOT7*, and *DCP2*, in both sequencing and quantitative PCR results when comparing Type 3 to Type 2 oocytes (Fig. 4f,g) [[69], [70], [71]]. This suggests that Type 3 oocytes prepare for and initiate the M-decay process. According to previously reported studies in humans and mice, to identify genes involved in the M-decay process, we compared MII-stage oocytes with Type 1 oocytes and defined genes with TPM > 2 and significantly downregulated in MII-stage oocytes (*p*-adjust ≤ 0.05 and log2FoldChange ≤ −0.5) as M-decay genes [68] (Fig. S4b). Furthermore, we compared Type 3 oocytes with Type 1 oocytes and applied the same criteria to define decay-associated genes in Type 3 oocytes for subsequent analysis (Fig. S4c). We observed 3976 decayed genes in MII-stage oocytes and 3465 decayed genes in Type 3 oocytes (Fig. S4b,c). Interestingly, we observed that most of the genes (2867/3465) decayed in Type 3 oocytes are also included in the M-decay gene set of MII-stage oocytes. Genes that undergo decay in Type 3 oocytes and are part of the M-decay gene set include those previously reported in the human M-decay process, such as *ZAR1, TLE6, PABPC1L, SIRT6*, and *AK2* [68] (Fig. 4h). Therefore, we infer that in the M-decay process of sheep, some oocytes progressing toward maturation, though still at the GV stage, have already prepared key M-decay factors and may initiate early degradation of a large portion of maternal mRNAs. This premature completion of most of the M-decay process at the GV stage may play an important role in the ovine oocyte fate determination.

### 3.6 The interaction of granulosa cell with oocytes during the maturation of antral follicles

The maturation process of follicles is intricately linked to the reciprocal interaction between granulosa cells and oocytes. A close relationship exists between oocyte maturation fate and granulosa cells. Investigating the communication between granulosa cells and oocytes may provide valuable insights into the external mechanisms that promote oocyte maturation. UMAP dimensionality reduction and clustering for cumulus and mural granulosa cells were firstly conducted. Unlike oocytes, these granulosa cell populations did not exhibit distinct developmental trajectories (Fig. S5a). To further explore the regulatory features shared among cumulus granulosa cells, mural granulosa cells, and oocytes, we therefore gathered and merged all size categories of cumulus granulosa cells and mural granulosa cells for subsequent analysis.

**Fig. 5:**
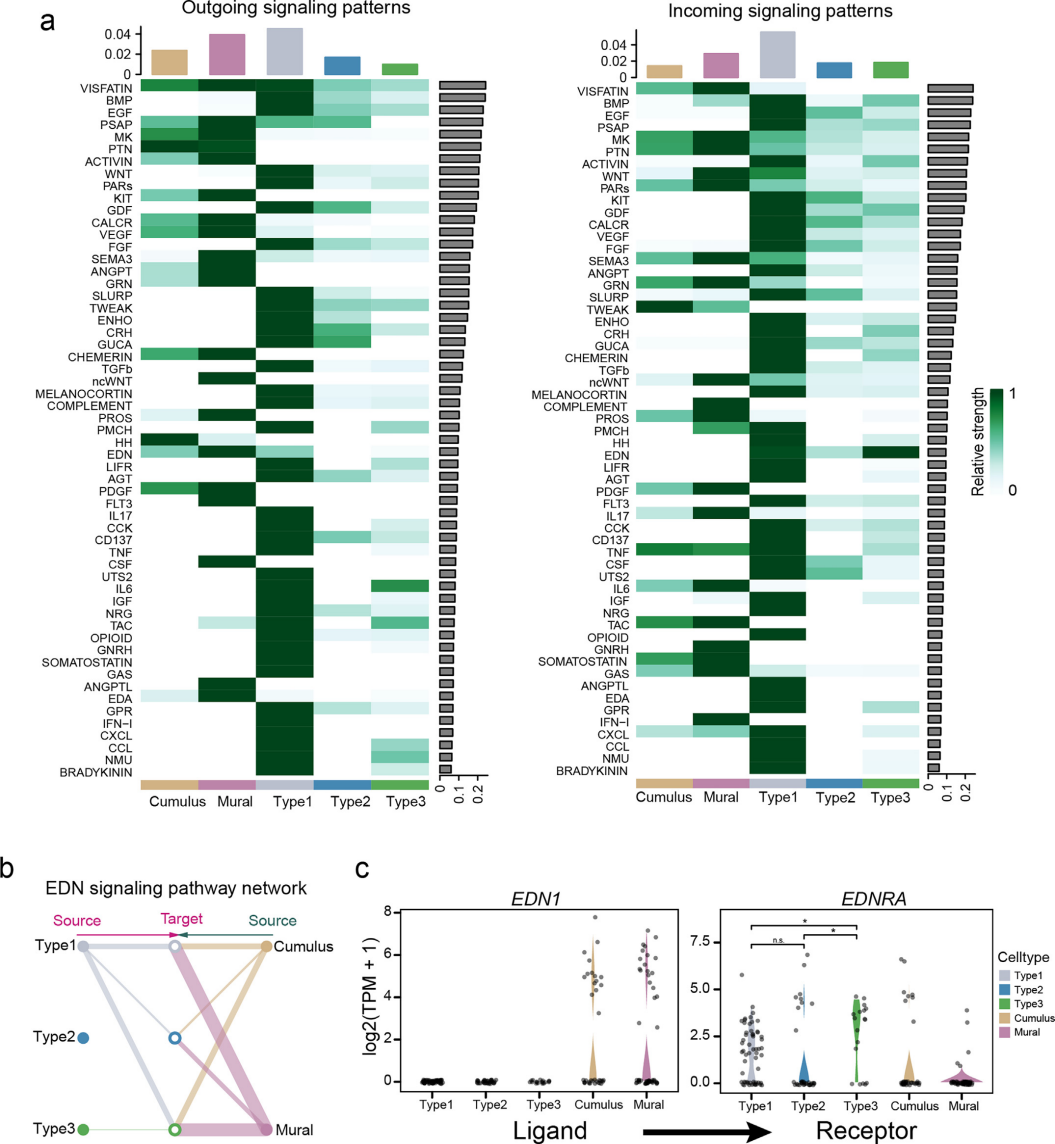
**Cell-cell communication between cumulus granulosa cells, mural granulosa cells, and the three types of oocytes.** (a) Heatmap showing outgoing and incoming pathway scores for signaling between cumulus granulosa cells, mural granulosa cells, and the three types of oocytes. The colored bars at the top represent the total signal strength across all pathways within each cell type. The color gradient in the heatmap reflects the relative signal strength of each signaling pathway across different cell types (row-scaled). The gray bars on the right indicate the total signal strength of each signaling pathway across all cell types. (b) Reception relationships and weights of the EDN signaling pathway network among cumulus granulosa cells, mural granulosa cells, and the three types of oocytes. (c) Expression profiles of *EDN1* (ligand) and *EDNRA* (receptor) in the EDN signaling pathway among cumulus granulosa cells, mural granulosa cells, and the three types of oocytes. *P < 0.05.

We then analyzed the cellular communication among cumulus granulosa cells, mural granulosa cells, and the three types of oocytes in antral follicles. The results indicated extensive cellular communication among the three types of oocytes, cumulus granulosa cells, and mural granulosa cells. Notably, granulosa cells exhibited more active communication with the earlier-stage Type 1 oocytes (Fig. 5a). Notably, we found that the EDN signaling pathway had a high enrichment score among the signaling pathways received by Type 3 oocytes (Fig. 5a). This pathway, also referred to as the endothelin signaling pathway, is intimately linked to the processes of follicle maturation and ovulation [72,73]. The endothelin signaling pathway comprises endothelin ligands and receptors. There are three types of endothelin ligands: Endothelin 1 (*EDN1*), Endothelin 2 (*EDN2*), and Endothelin 3 (*EDN3*). The endothelin receptors include two types: Endothelin Receptor Type A (*EDNRA*) and Endothelin Receptor Type B (*EDNRB*) [74]. Typically, *EDNRA* has a higher affinity for *EDN1* compared to the other two ligands, while *EDNRB* exhibits similar affinity for all three ligands [74]. Therefore, we first aim to identify which ligands and receptors play a primary role in the communication between granulosa cells and oocytes. The results indicate that among the three ligands, *EDN1* is predominantly expressed and is found exclusively in the two types of granulosa cells, consistent with previous studies that suggest endothelin signaling in the ovaries is derived from granulosa cells [73] (Figs. 5b,c and S5b,c). Between the two receptors, *EDNRA* is predominantly expressed, and its expression in Type 3 oocytes is significantly higher than in the other two types of oocytes (Figs. 5c and S5d). Based on the above data, we hypothesize that differences in endothelin signaling, such as the differential expression of the receptor *EDNRA* across oocyte types, may contribute to variations in their developmental fate. The above results will provide valuable insights for further exploration into how granulosa cells contribute to the ovine developmental fate of different oocytes in subsequent studies. Next, we will focus on the endothelin signaling pathway and the receptor *EDNRA*, conducting corresponding experimental validation.

### 3.7 Roles of EDN1/EDNRA signaling in development potential of sheep oocytes

**Fig. 6:**
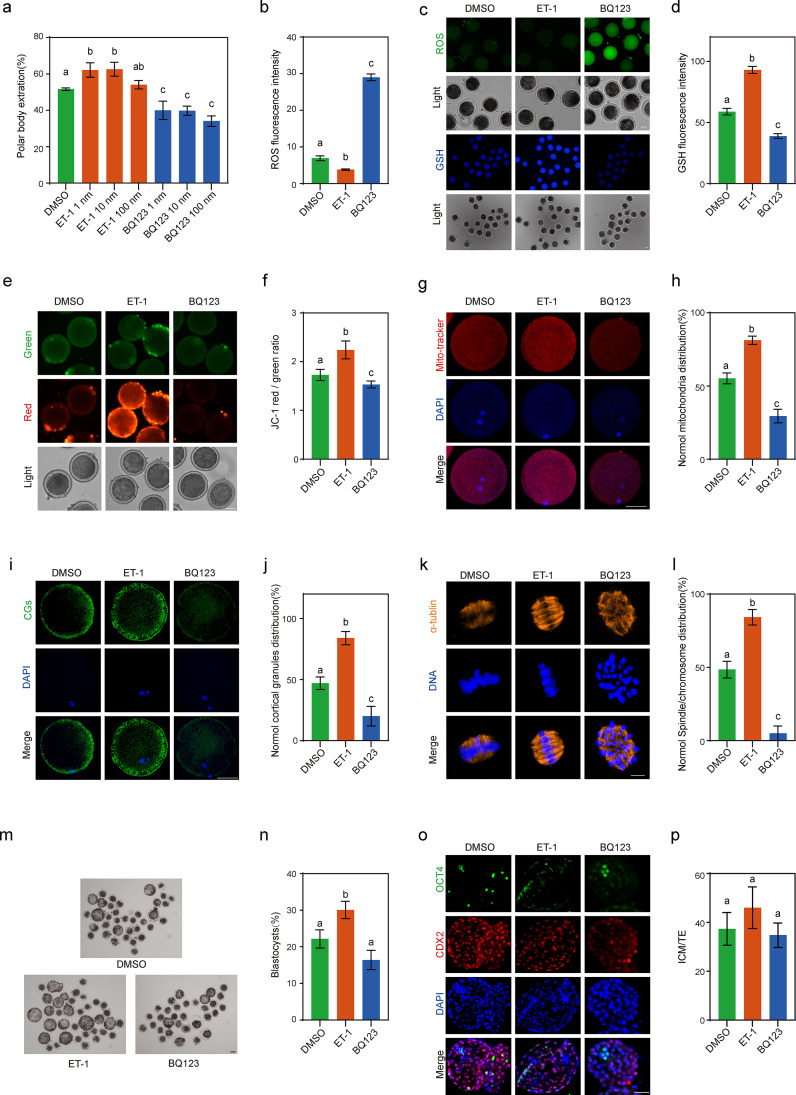
**Modulating sheep oocyte developmental fate through ET-1 supplement.** (a) The rate of polar body extrusion after 18 h IVM in ET-1, DMSO, BQ123. (b) The fluorescence intensity of ROS signals was compared in ET-1, DMSO, and BQ123. (c) Representative images of ROS levels of mature oocytes in the ET-1, DMSO, BQ123. Scale bar, 50 µm. Representative images of GSH levels of mature oocytes in the ET-1, DMSO, BQ123. Scale bar, 100 µm. (d) The fluorescence intensity of GSH signals was compared in ET-1, DMSO, and BQ123. (e) Representative images of Mitochondrial membrane potential (ΔΨm), detected by JC-1 staining in ET-1, DMSO, BQ123. (f) The ratio of red to green fluorescence intensity indicates the mitochondrial membrane potential level in the ET-1, DMSO, BQ123. (g) Representative images of the mitochondrial distribution of mature oocytes in the ET-1, DMSO, BQ123, detected by Mito-Tracker Red. Scale bar = 50 µm. (h) The rate of abnormal mitochondrial distribution in ET-1, DMSO, BQ123. (i) Representative images of the CGs distribution of an equatorial section of mature oocytes in the ET-1, DMSO, BQ123, detected by FITC-PNA. Scale bar = 50 µm. (j) The rate of normal CGs distribution in ET-1, DMSO, BQ123. (k) Representative images of the spindle morphology and chromosome alignment of mature oocytes in the ET-1, DMSO, BQ123, detected by immunofluorescent staining. Scale bar = 5 µm. (l) The rate of normal Spindle/chromosome distribution in ET-1, DMSO, BQ123. (m) Representative images of the blastocyst after 7.5 dpi IVF of ET-1, DMSO, BQ123. (n) The rate of blastocyst formation at 7.5 dpi was compared in ET-1, DMSO, and BQ123. (o) Representative images of immunofluorescent staining of CDX2 and OCT4 in blastocysts in ET-1, DMSO, BQ123, with DAPI for DNA. Scale bar = 50 µm. (p) The ratio of Blastocysts’ ICM/TE was compared in ET-1, DMSO, and BQ123. Letters that are the same indicate no significant difference (*P* > 0.05), while different letters indicate significant differences (*P* ≤ 0.05).

As shown in the above-mentioned analysis results, the endothelin signaling is an important link between granulosa cells and oocytes. From the sequencing data, we observed that *EDN1* is dominantly expressed in the two types of granulosa cells, and the receptor *EDNRA* exhibited differential expression level in oocytes of the three types. The expression pattern of *EDN1* and *EDNRA* indicated that *EDN1*/*EDNRA* signaling is involved in the development of sheep oocytes from antral follicles. According to previous studies, *EDN1* is ET-1–protein-encoding gene and secreted *ET-1* protein. BQ123 serves as antagonist of *EDNRA* [73,75]. Thus, to further explore the role of *EDN1*/*EDNRA* signaling in developmental potential of sheep oocytes, the ovine oocytes from antral follicles were cultured in maturation medium supplemented with different concentrations of ET-1 and BQ123, DMSO was used as a control. And then the first polar body (PB1) extrusion rates were calculated, which is a key indicator of nuclear maturation. The results showed that 10 nM ET-1 exhibited a significant increase in the PB1 extrusion rate, whereas BQ123 group, all three concentrations (1 nM, 10 nM, 100 nM) showed similar significant decrease patterns in the PB1 extrusion rate (Fig. 6a) (DMSO: 51.68 ± 0.69; ET-1 1 nM: 62.16 ± 3.93; ET-1 10 nM: 62.62 ± 3.82; ET-1 100 nM: 54.10 ± 2.33; BQ123 1 nM: 40.04 ± 5.08; BQ123 10 nM: 39.80 ± 2.60; BQ123 100 nM: 34.07 ± 2.88).

Based on the above results, we focused on examining the characteristics of matured oocytes from 10 nM ET-1 and 10 nM BQ123 treatments, respectively. It is acknowledged that oxidative stress is one of the main factors attributed to the decreased quality of in vitro matured oocytes. The ROS and GSH levels of 3 types of oocytes (DMSO, ET-1 and BQ123) were detected using DCFH and Cell Tracker Blue, respectively. The ROS level in the ET-1 group (3.80 ± 0.20) was significantly lower than the control group (6.90 ± 0.66), whereas the ROS content in the BQ123 (29.01 ± 0.94) was significantly higher than the control group (Fig. 6b,c). Meanwhile, the oocytes of ET-1 (93.14 ± 2.92) and BQ123 (58.89 ± 2.63) treatments showed increased and decreased GSH level, respectively, compared to the DMSO group (38.88 ± 1.94) (Fig. 6c,d).

It is well-known that the redistribution of cytoplasmic organelles is essential for the oocyte which matures in vitro [76]. Therefore, We the distribution of mitochondria, the endoplasmic reticulum (ER), Golgi apparatus, and cortical granules in IVM sheep oocytes from ET-1, BQ123, and DMSO treatment were further measured. Mitochondria are the key indicators of oocyte quality [77]. The membrane potential can directly reflect the function of mitochondria. Thus, the mitochondrial membrane potential were analyzed by JC-1 staining and the results indicated that ET-1 treatment enhanced the mitochondrial membrane potential of oocytes from in vitro maturation (DMSO: 1.73 ± 0.11; ET-1: 2.24 ± 0.18; BQ123: 1.53 ± 0.07) (Fig. 6e,f). Secondly, the distribution patterns of mitochondria were further determined by Mito-Tracker staining (Fig. 6g). Mitochondria in the ET-1 group (81.25% ± 2.78%) revealed a homogeneous distribution in the cytoplasm, and the ratio of normal mitochondria distribution was significantly higher than the control group (55.25% ± 3.68%). In contrast, in the BQ123 group (29.50% ± 4.60%), most of the mitochondrial signals were not uniformly distributed and had the lowest ratio of normal mitochondria distribution (Fig. 6h). At last, the ATP contents were measured and ET-1 treatment (0.32 ± 0.02) was significantly higher than the control (0.17 ± 0.02) and BQ123 groups (0.13 ± 0.01) (Fig. S6a). These results suggest that the IVM oocyte mitochondrial dysfunction could be optimized by ET-1 supplementation.

Next, we detected the distribution of the ER and Golgi apparatus. ET-1 supplementation could significantly increase the proportion of ER’s normal distribution in mature oocytes, while the rate of normal ER distribution was significantly decreased in BQ123 treatment (DMSO: 60.25% ± 4.19%; ET-1: 82.5% ± 0.01%; BQ123: 30.5% ± 9.22%) (Fig. S6b). Similarly, the Golgi-tracker and statistical analysis results showed that ET-1 supplementation significantly increased the rate of normal Golgi distribution, whereas BQ123 had the opposite result (DMSO: 62.00% ± 9.29%; ET-1: 76.33% ± 4.80%; BQ123: 41.33% ± 4.37%) (Fig. S6c).

And then we examined the distribution of the cortical granules (CGs), which is one of the important indicators of oocyte cytoplasmic maturation and is associated with the blockade of polyspermy following fertilization. Lens culinaris agglutinin (LCA)-FITC staining results showed that after ET-1 supplementation, the proportion of oocytes with cortical granule normal distribution was significantly higher compared to the control group (Fig. 6i). In contrast, after BQ123 treatment, the proportion of oocytes with normal distribution was significantly reduced (DMSO: 47.00% ± 5.196%; ET-1: 84.00% ± 5.416%; BQ123: 20.00% ± 8.031%) (Fig. 6j).

Moreover, abnormal spindle/chromosome arrangement can lead to subsequent aneuploidy. The spindle morphology and chromosome alignment in matured oocytes were also evaluated by immunofluorescence staining and observed by Confocal microscopy. The results showed that a typical barrel-shaped spindle and well-aligned chromosomes at the equator in the group with ET-1, and the percentage of oocytes with normal spindle-chromosome complexes significantly increased compared to the control and BQ123 groups (DMSO: 48.5% ± 5.679%; ET-1: 84.25% ± 5.297%; BQ123: 5% ± 0.05%). In conclusion, the addition of ET-1 during the process of in vitro maturation promoted the formation of normal spindle (Fig. 6k,l).

Above all, the 10 nM ET-1 and BQ123 were used to further in vitro fertilization and culture assays with an equal number of MII-stage oocytes (IVM obtained), which showed that oocytes treated with ET-1 achieved a higher blastocyst rate (30% ± 2.393%) compared to the control group (22.15% ± 2.5%) (Fig. 6m,n). Meanwhile, the blastocyst rate significantly decreased in the BQ123 group (16.42% ± 2.62%). Subsequently, to check the quality of resultant blastocysts, ICM and TE from control, ET-1 and BQ123 groups were co-immunostained using OCT3/4 (ICM marker) and CDX2 (TE marker) antibodies. Using images obtained from the confocal microscope, the ratio of ICM/TE cells was counted. A moderately higher ratio was observed in the ET-1 group compared to the other groups, but this difference did not reach statistical significance (Fig. 6o,p).

## 4 Discussion

**Fig. 7:**
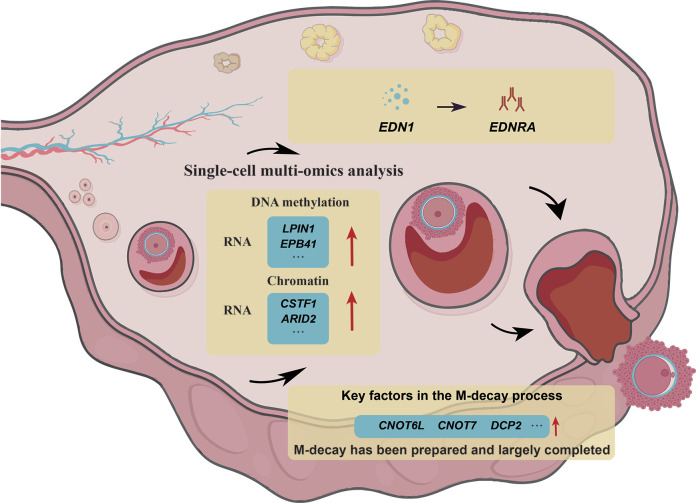
**Diagram of oocyte fate trajectory in ovine antral follicles.** The maturation of oocytes in ovine antral follicles is orchestrated through distinct regulatory pathways. One of these pathways involves the granulosa cells, which provide the ET-1 ligand during oocyte development. The oocyte expresses the *EDNRA*, receiving the ET-1 signal, which promotes its progression toward maturation. Additionally, Elevated expression of these M-decay factors (such as *CNOT6L, CNOT7* and *DCP2*) facilitates the preparation and near-completion of the M-decay process in ovine oocytes, advancing toward maturity. Meanwhile, these upregulated genes, modulated by DNA methylation dynamics (such as *LPIN1* and *EPB41*) and chromatin accessibility (such as *CSTF1* and *ARID2*), play essential regulatory roles during oocyte developmental progression.

The acquisition of fully competent oocytes is essential for successful in vitro embryo production. Achieving this requires a comprehensive understanding of the developmental mechanisms of oocytes *in vivo*. As antral follicles develop *in vivo*, the oocytes are subject to two potential fates: atresia or maturation. The complex regulatory mechanisms governing the fate of oocytes towards maturation during *in vivo* antral follicle development remain poorly understood. In this study, we collected sheep oocytes, cumulus granulosa cells, and mural granulosa cells from antral follicles of varying sizes, and employed our modified single-cell multi-omics sequencing technology to comprehensively analyze the molecular characteristics of oocyte growth and maturation during the antral follicle development process in sheep. We acquired high-quality transcriptomic, DNA methylation, and chromatin accessibility data from oocytes of varying follicle sizes, enabling us to dissect the multi-layered regulatory mechanisms underlying oocyte maturation fate (Fig. 7).

Using MII-stage oocytes as a reference to trace the potential developmental fate of oocytes from different-sized antral follicles, we performed pseudotime analysis to reclassify all antral follicle oocytes into three types: Type 1, corresponding to an earlier developmental stage; Type 3, representing oocytes progressing towards maturation; and Type 2, consisting of oocytes failing to reach maturation. Notably, Type 3 oocytes exhibited a developmental profile akin to MII-stage oocytes, both at the transcriptomic and epigenomic levels.

To further investigate the differences between oocytes with distinct developmental fates, we focused on a direct comparison between Type 3 and Type 2 oocytes. Our analysis revealed differential expression patterns of general transcription factors between the two oocyte types. Notably, the general transcription factor TFIIA, which plays a crucial role in the clearance of maternal mRNA, was significantly upregulated in Type 3 oocytes. Maternal mRNA degradation is a pivotal step in the transition from maternal control to zygotic control (MZT) and is typically divided into two pathways: maternal (M) decay and zygotic (Z) decay [1]. The former is mediated by maternal factors accumulated in the mature oocyte, while the latter relies on zygotic transcripts produced following fertilization [67]. Relevant studies have reported that in human oocytes, proteins associated with M-decay, such as *CNOT7*, are barely detectable at the GV stage but accumulate in mature oocytes, with M-decay being largely completed by the MII stage [68]. Our study also revealed a significant enrichment of the mRNA clearance process in Type 3 oocytes, with key factors known to regulate M-decay, such as *CNOT6L, CNOT7*, and *DCP2*, showing markedly higher expression in Type 3 oocytes. These findings indicate that the M-decay process is likely initiated in Type 3 oocytes. Compared to the mRNA degraded in MII-stage oocytes, the mRNA degraded in Type 3 oocytes almost entirely overlaps with it, accounting for a large proportion of the degradation products. Previous studies have indicated that oocyte maturation involves adenylate-dependent degradation, and the decline in MII-stage oocyte quality during in vitro culture is closely related to de-adenylation in the maternal mRNA clearance process [78,79]. Based on our findings, at the antral follicle stage, GV oocytes, especially Type 3 oocytes have already begun the preparatory steps for the M-decay process, which is largely completed before meiosis. The signaling pathways responsible for activating the key genes involved in M-decay and the causal relationship between the initiation of M-decay and the maturation fate of GV-stage oocytes warrant in-depth research.

Granulosa cells play a pivotal role in antral follicle growth and oocyte maturation. Therefore, we analyzed the cell-cell communication between cumulus granulosa cells, mural granulosa cells, and the three types of oocytes. Specifically, we found that Type 3 oocytes exhibited strong enrichment for signal reception components of the BMP, ACTIVIN, CRH, CHEMERIN, and EDN signaling pathways. This suggests a potential close association between these pathways and oocyte maturation (Fig. 5a). Notably, the EDN (endothelin) signaling pathway displayed the most pronounced enrichment in Type 3 oocytes. Based on this prominence, we investigated this pathway in greater detail. The endothelin signaling pathway consists of three endothelin ligands—*EDN1, EDN2*, and *EDN3*—and two receptors, *EDNRA* and *EDNRB*, and is closely associated with angiogenesis [80]. Moreover, it has been reported that ET-1 plays a role in promoting oocyte maturation in humans [73]. Through data analysis, we found that the ligand *EDN1*, produced by cumulus and mural granulosa cells, is highly expressed in Type 3 oocytes that are on the path to maturation, which also exhibits elevated levels of the receptor *EDNRA*. Through experimental validation, we demonstrated that the addition of the hormone ET-1 to in vitro cultured oocytes enhances the quality of key organelles, such as mitochondria, the Golgi apparatus, and the endoplasmic reticulum, while also improving spindle organization. However, when treated with the *EDNRA* inhibitor BQ123, these critical organelles and spindle alignment were significantly disrupted. In subsequent in vitro fertilization and embryo culture assays, oocytes supplemented with ET-1 showed a significant increase in blastocyst formation rate whereas BQ123 reduced the blastocyst production. These results suggest that the enhanced endothelin signaling (*EDN1/EDNRA*) plays a crucial role in oocyte maturation and improves the developmental potential of oocytes. Our results are similar to those observed in humans; however, in mice, endothelin-1 has no effect on the extrusion of the first polar body or cytoplasmic maturation of pre-ovulatory oocytes [73]. This discrepancy may be attributed to species differences. In this regard, sheep may serve as a valuable model for studying human oocyte maturation. However, further studies still require more refined and advanced experiments to explore the exact mechanisms underlying the regulation of the endothelin signaling pathway between oocytes and granulosa cells.

## 5 Conclusion

Overall, the current study decoded the multi-omics characteristics of sheep oocytes *in vivo* at single-cell level, and revealed the molecular features of GV oocytes with distinct fates during the process of antral follicles growth *in vivo*. Through comparison, we found that GV oocytes with a trend toward maturation prepared for the M-decay process and completed much of the maternal mRNA degradation. By examining the communication between oocytes and granulosa cells, we identified the endothelin signaling pathway (*EDN1/EDNRA*) and further demonstrated experimentally that ET-1 promotes the maturation and quality of sheep GV-stage oocytes in vitro. These findings offer new insights into the determination of mammalian oocyte fate and hold significant implications for in vitro embryo production.

## For studies with animals

Ethical approval: Approval to conduct this study was obtained from the Animal Care Committee of China Agricultural University before the beginning of animal experiments (Ethics approval number: AW41015202-1-04).

## CRediT authorship contribution statement

**Yujun Yao:** Data curation. **Zihuan Du:** Validation. **Qiang Zhang:** Resources. **Xiaochen Kou:** Validation. **Hongyu Yang:** Validation. **Chanyuan Pan:** Resources. **Jing Li:** Resources. **Xiangwei Sun:** Validation. **Lu Zhang:** Supervision. **Guoshi Liu:** Supervision. **Xiaoling Xu:** Methodology. **Shuai Gao:** Methodology.

## Declaration of competing interest

The authors declare that they have no conflicts of interest in this work.

## Appendix Supplementary materials

**Fig. S1. Sequencing data statistics for oocytes, cumulus granulosa cells, and mural granulosa cells.** (a) Metrics for raw sequencing data of oocytes, cumulus granulosa cells, and mural granulosa cells are shown. Each point represents the mean for various parameters, with whiskers indicating the maximum and minimum values. (b) Heatmap of Pearson correlation coefficients between individual oocytes. (c) Expression patterns of oocyte marker genes in oocytes derived from follicles of different sizes or developmental stages. Image, image 1 dummy alt text

**Fig. S2. Pseudotime analysis of oocyte transcriptomic data. (**a) Pseudotime trajectory plot illustrating the distribution of oocytes from three follicle sizes and MII-stage oocytes. (b) Proportion plot showing the distribution of oocytes from different follicle sizes and MII-stage oocytes across the three oocyte types. Image, image 2 dummy alt text

**Fig. S3. Analysis of DNA Methylation and Chromatin Accessibility in Oocytes.** (a) Boxplot showing DNA methylation levels of three oocyte types. (b) Boxplot showing chromatin accessibility levels of the three oocyte types. ***P* < 0.01. (c) Enrichment scores of NDRs in various gene elements for different oocyte types and MII-stage oocytes. (d) Motif enrichment analysis of distal-NDRs in the three oocyte types. (e) Motif enrichment analysis of proximal NDRs in Type 1 oocytes. The value in parentheses represents the enrichment P-value, and the vertical axis represents the conservation score of each base. (f) Motif enrichment analysis of proximal NDRs in Type 2 oocytes. (g) Motif enrichment analysis of proximal NDRs in Type 3 oocytes. The value in parentheses represents the enrichment *P*-value, and the vertical axis represents the conservation score of each base. Image, image 3 dummy alt text

**Fig. S4. Comparative analysis of Type 2 and Type 3 oocytes.** (a) Expression levels of general transcription factors in Type 2 and Type 3 oocytes. *****P* < 0.0001, ****P* < 0.001, ***P* < 0.01. (b) Volcano plot of decayed genes in MII-stage oocytes compared to Type 1 oocytes. (c) Volcano plot of decayed genes in Type 3 oocytes compared to Type 1 oocytes. Image, image 4 dummy alt text

**Fig. S5. Granulosa Cell Analysis and Cell–Cell Communication.** (a) UMAP plot of integrated cumulus and mural granulosa cells from follicles of different sizes. Colors indicate follicle size or developmental stage, and point shapes represent granulosa cell subtypes. (b) Expression levels of *EDN2* across three oocyte types and two granulosa cell types. (c) Expression levels of *EDN3* across three oocyte types and two granulosa cell types. ****P* < 0.001. (d) Expression levels of *EDNRB* across three oocyte types and two granulosa cell types. **P* < 0.05. Image, image 5 dummy alt text

**Fig. S6. Modulating sheep oocyte developmental fate through ET-1 supplement.** (a) The rate of average relative ATP was compared in ET-1, DMSO, BQ123 (pm). (b) Representative images of the endoplasmic reticulum distribution of mature oocytes in ET-1, DMSO, BQ123, detected by ER-Tracker Green. Scale bar  =  50 µm. The rate of normal endoplasmic reticulum distribution in ET-1, DMSO, BQ123. (c) Representative images of the Golgi apparatus distribution of mature oocytes in ET-1, DMSO, BQ123, detected by Golgi-Tracker Red. Scale bar  =  50 µm. The rate of normal Golgi apparatus distribution in ET-1, DMSO, BQ123. (d) The rate of cleavage formationat 2 dpi was compared in ET-1, DMSO, BQ123. Image, image 6 dummy alt text

Image, application 7

Image, application 8

Image, application 9

Image, application 10
