# Supplementary figures and images for: Single-cell multi-omics analysis decodes molecular characteristics of sheep oocyte fate in vivo maturation
Source: Fundam Res. 2025 Dec 8;6(3):1578–94. doi: 10.1016/j.fmre.2025.10.009 (PMC13247468; doi:10.1016/j.fmre.2025.10.009)

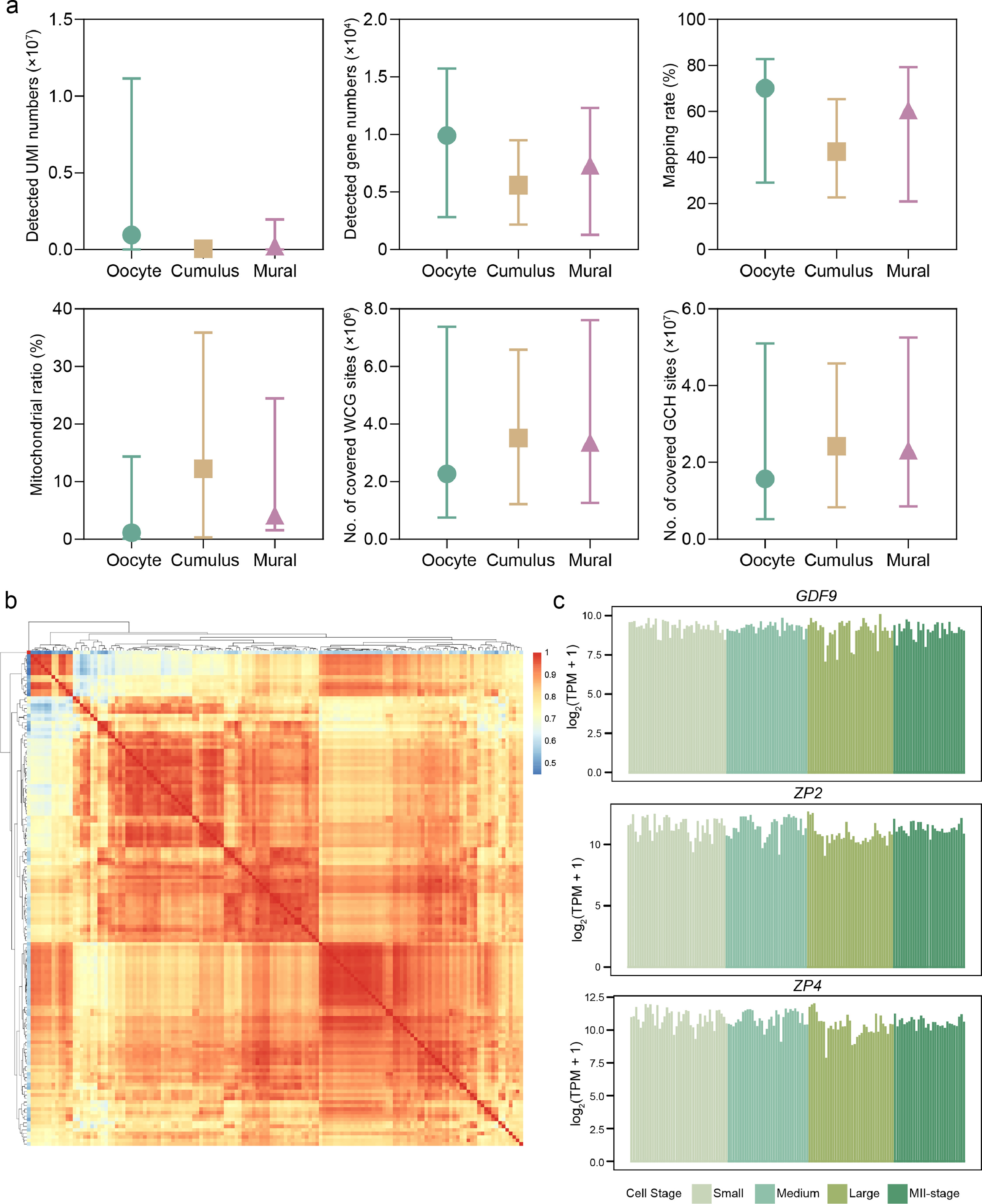

Supplement: Supplementary file 1 — Fig. S1. Sequencing data statistics for oocytes, cumulus granulosa cells, and mural granulosa cells. (a) Metrics for raw sequencing data of oocytes, cumulus granulosa cells, and mural granulosa cells are shown. Each point represents the mean for various parameters, with whiskers indicating the maximum and minimum values. (b) Heatmap of Pearson correlation coefficients between individual oocytes. (c) Expression patterns of oocyte marker genes in oocytes derived from follicles of different sizes or developmental stages. [file mmc1.jpg]

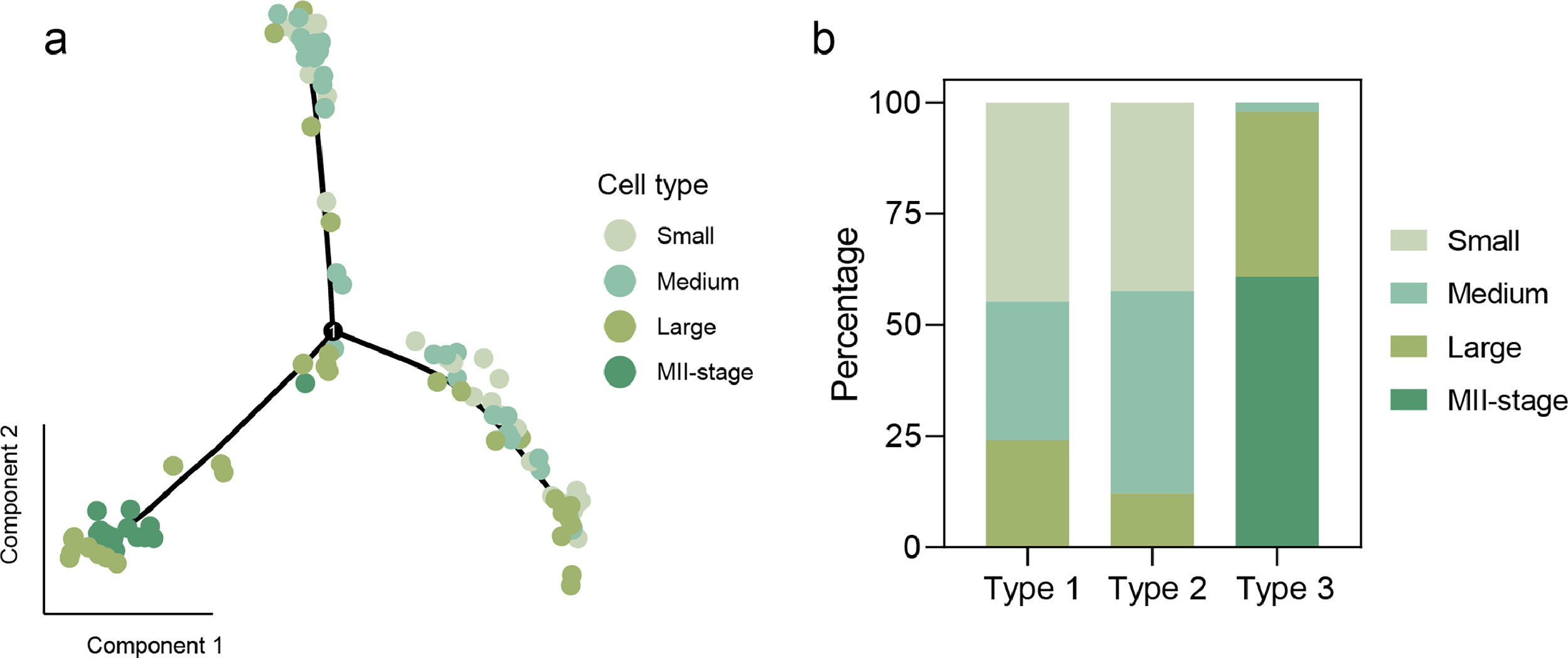

Supplement: Supplementary file 2 — Fig. S2. Pseudotime analysis of oocyte transcriptomic data. (a) Pseudotime trajectory plot illustrating the distribution of oocytes from three follicle sizes and MII-stage oocytes. (b) Proportion plot showing the distribution of oocytes from different follicle sizes and MII-stage oocytes across the three oocyte types. [file mmc2.jpg]

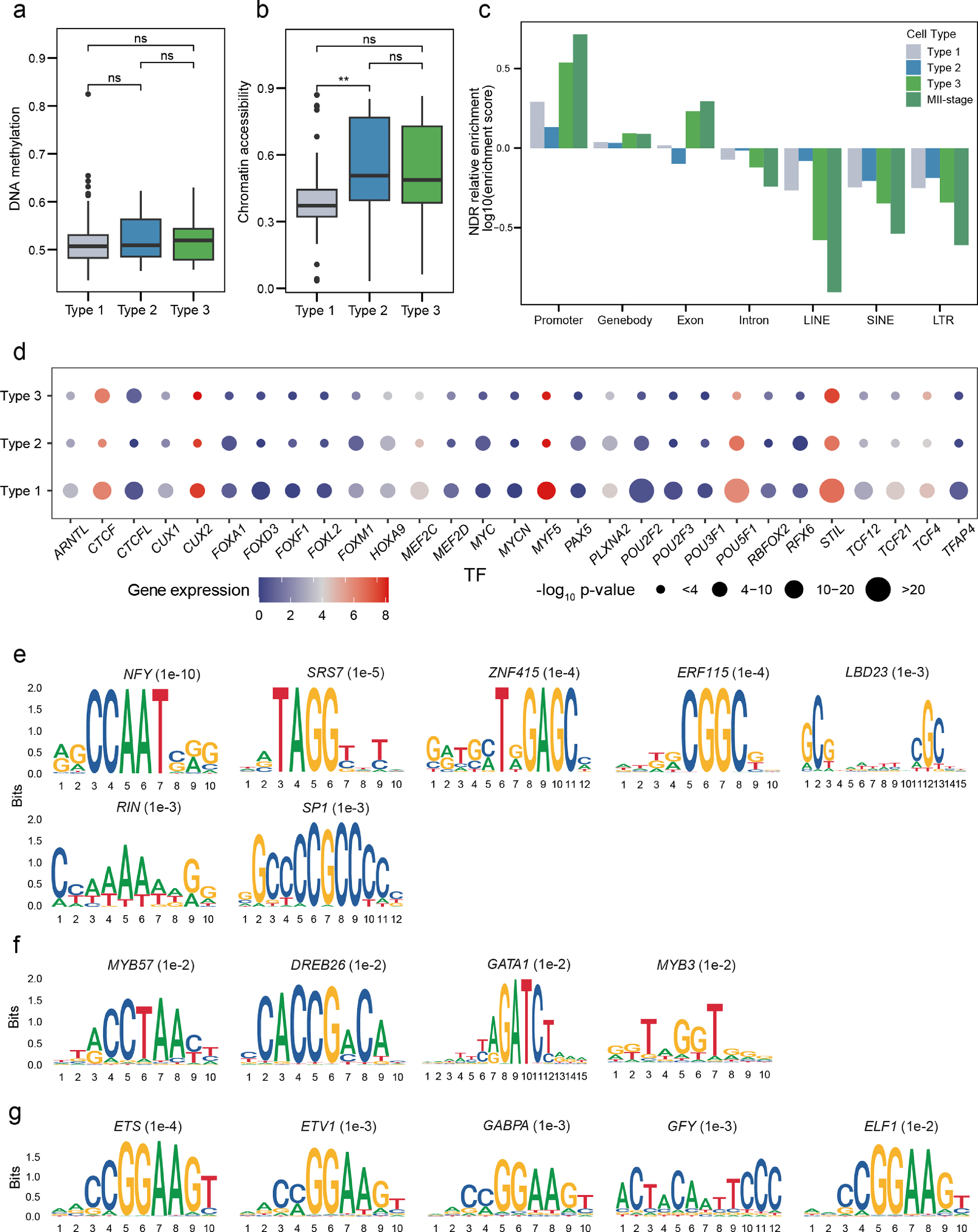

Supplement: Supplementary file 3 — Fig. S3. Analysis of DNA Methylation and Chromatin Accessibility in Oocytes. (a) Boxplot showing DNA methylation levels of three oocyte types. (b) Boxplot showing chromatin accessibility levels of the three oocyte types. **P < 0.01. (c) Enrichment scores of NDRs in various gene elements for different oocyte types and MII-stage oocytes. (d) Motif enrichment analysis of distal-NDRs in the three oocyte types. (e) Motif enrichment analysis of proximal NDRs in Type 1 oocytes. The value in parentheses represents the enrichment P-value, and the vertical axis represents the conservation score of each base. (f) Motif enrichment analysis of proximal NDRs in Type 2 oocytes. (g) Motif enrichment analysis of proximal NDRs in Type 3 oocytes. The value in parentheses represents the enrichment P-value, and the vertical axis represents the conservation score of each base. [file mmc3.jpg]

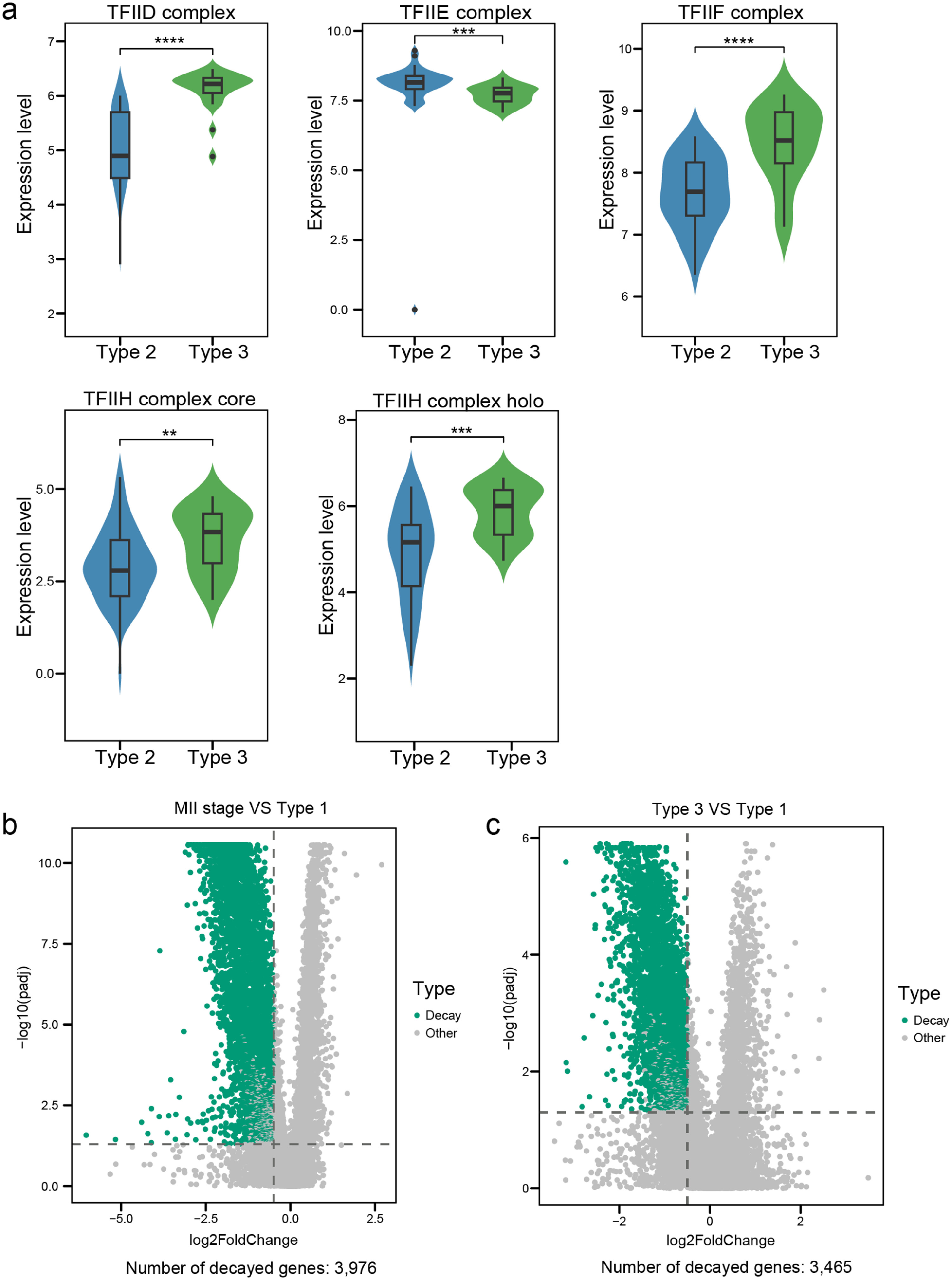

Supplement: Supplementary file 4 — Fig. S4. Comparative analysis of Type 2 and Type 3 oocytes. (a) Expression levels of general transcription factors in Type 2 and Type 3 oocytes. ****P < 0.0001, ***P < 0.001, **P < 0.01. (b) Volcano plot of decayed genes in MII-stage oocytes compared to Type 1 oocytes. (c) Volcano plot of decayed genes in Type 3 oocytes compared to Type 1 oocytes. [file mmc4.jpg]

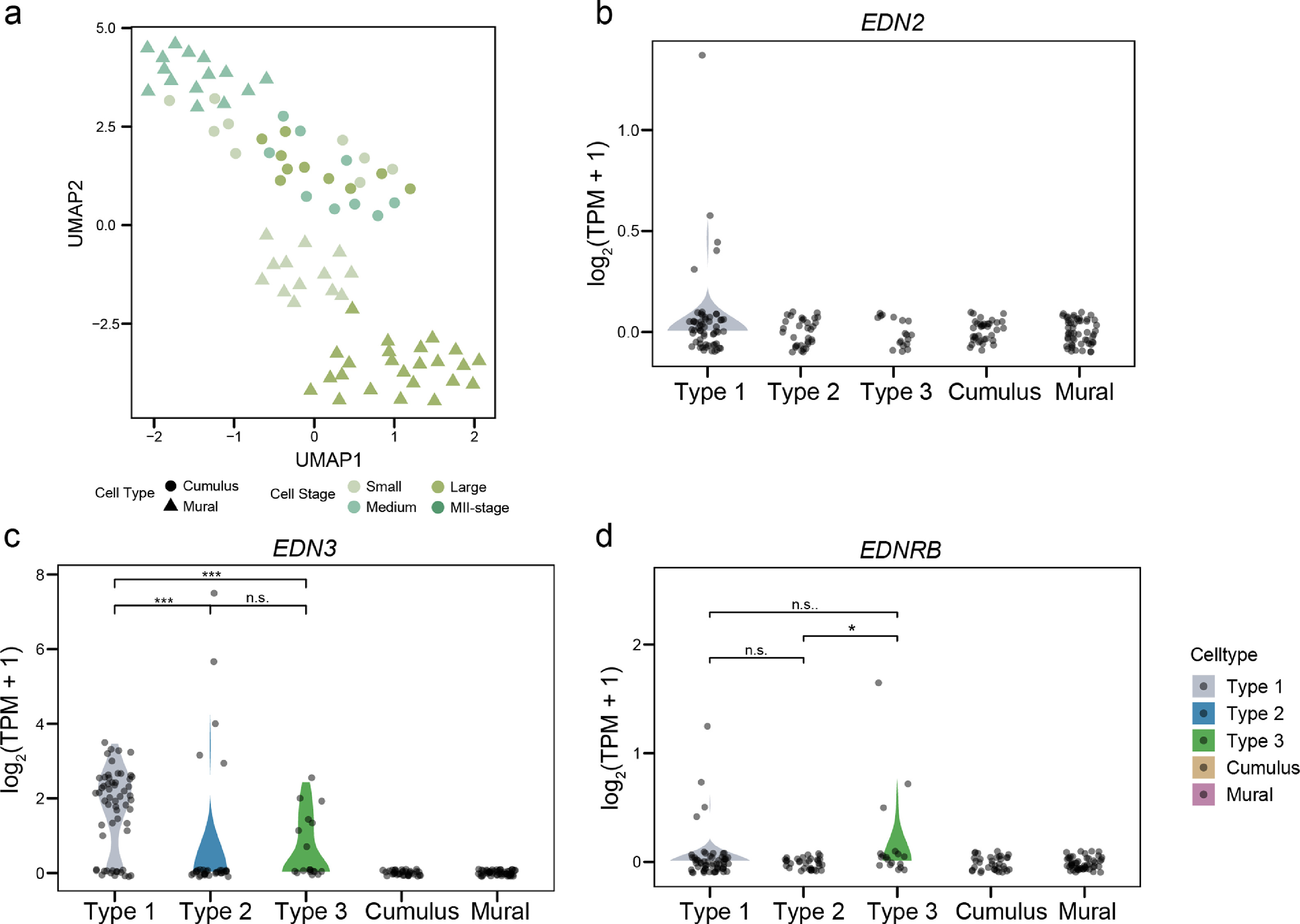

Supplement: Supplementary file 5 — Fig. S5. Granulosa Cell Analysis and Cell–Cell Communication. (a) UMAP plot of integrated cumulus and mural granulosa cells from follicles of different sizes. Colors indicate follicle size or developmental stage, and point shapes represent granulosa cell subtypes. (b) Expression levels of EDN2 across three oocyte types and two granulosa cell types. (c) Expression levels of EDN3 across three oocyte types and two granulosa cell types. ***P < 0.001. (d) Expression levels of EDNRB across three oocyte types and two granulosa cell types. *P < 0.05. [file mmc5.jpg]

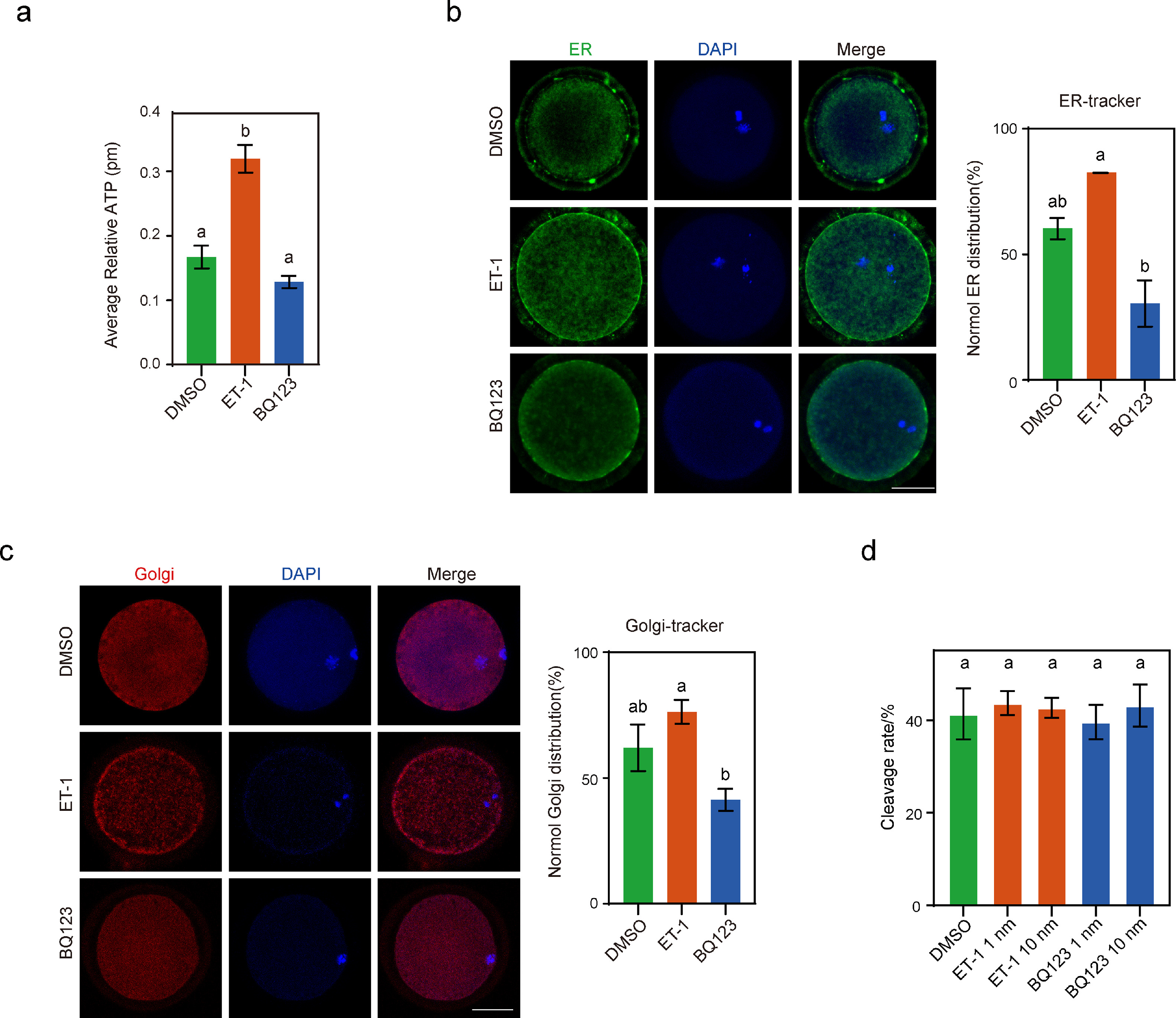

Supplement: Supplementary file 6 — Fig. S6. Modulating sheep oocyte developmental fate through ET-1 supplement. (a) The rate of average relative ATP was compared in ET-1, DMSO, BQ123 (pm). (b) Representative images of the endoplasmic reticulum distribution of mature oocytes in ET-1, DMSO, BQ123, detected by ER-Tracker Green. Scale bar = 50 µm. The rate of normal endoplasmic reticulum distribution in ET-1, DMSO, BQ123. (c) Representative images of the Golgi apparatus distribution of mature oocytes in ET-1, DMSO, BQ123, detected by Golgi-Tracker Red. Scale bar = 50 µm. The rate of normal Golgi apparatus distribution in ET-1, DMSO, BQ123. (d) The rate of cleavage formationat 2 dpi was compared in ET-1, DMSO, BQ123. [file mmc6.jpg]
