# Supplementary material for: Single-cell multi-omics analysis decodes molecular characteristics of sheep oocyte fate in vivo maturation
Source: Fundam Res. 2025 Dec 8;6(3):1578–94. doi: 10.1016/j.fmre.2025.10.009 (PMC13247468; doi:10.1016/j.fmre.2025.10.009)
Supplement: Supplementary file 10 [file mmc10.docx]

| tet1-F | AATCTGTCCGGAACACGAGG |
| --- | --- |
| tet1-R | GTGACCCCAGAGAAAGGACG |
| tet2-F | GAGCAGGTCCTAATGTGGCA |
| tet2-R | CTTGTCGGCCAGAGAGACTG |
| tet3-F | GCATGAATGGGGATTTGGCA |
| tet3-R | CCAGACAAGCTCCCAGTCTC |
| btg4-F | GAGGAACTGGTTTGCTTGGC |
| btg4-R | AGCCTACGAGGAGGTCTGAA |
| zar1-F | GACTGCAATATCCGCTGGGA |
| zar1-R | GCTGTCACAGGATAGGCGTT |
| dnmt1-F | CACTTTCTTTGCGGACAAATTGAGG |
| dnmt1-R | TGCTTCTTTTTCTTCCCCTGGT |
| CNOT7-2-F | TCACCTGGAGTGTGAGTTCC |
| CNOT7-2-R | GGGCACAAGGAGTCTAGCTG |
| CNOT7-F | AGTGTGAGTTCCGGAGGGAC |
| CNOT7-R | AAGGAGTCTAGCTGCCAAGC |
| DCP2-F | GCTGGTAGCACACCAGCTAA |
| DCP2-R | CCTCTGTGCCCACATAGCAA |
| TAF4B-F | TGAAATTTCTGGCGGGGGAT |
| TAF4B-R | ACAGCATCGCTAGGGAGAGG |
| GTF2A2-F | GGGAGAGGTGGTCGGAAAAG |
| GTF2A2-R | GCCAGTGCTGCGTTTATAGC |
| GAPDH-F | GTGTCTGTTGTGGATCTGACCTG |
| GAPDH-R | AGAAGAGTGAGTGTCGCTGTTGAAGT |
| GTF2A1-3-F | AAGCCTGAAATGGGAGCGAT |
| GTF2A1-3-R | TCTGCATCTCCAATGGCTTT |
| GTF2A2-3-F | GGAGAGGTGGTCGGAAAAGTAG |
| GTF2A2-3-R | CCTCGGAGCGGACAGAAGTG |
| CNOT6L-8-F | CCAGAGGCATATGGTTTGGCA |
| CNOT6L-8-R | TCCTGGGACACAGTTGACTT |
